# Consumption of Dairy Products and Colorectal Cancer in the European Prospective Investigation into Cancer and Nutrition (EPIC)

**DOI:** 10.1371/journal.pone.0072715

**Published:** 2013-09-02

**Authors:** Neil Murphy, Teresa Norat, Pietro Ferrari, Mazda Jenab, Bas Bueno-de-Mesquita, Guri Skeie, Anja Olsen, Anne Tjønneland, Christina C. Dahm, Kim Overvad, Marie Christine Boutron-Ruault, Françoise Clavel-Chapelon, Laura Nailler, Rudolf Kaaks, Birgit Teucher, Heiner Boeing, Manuela M. Bergmann, Antonia Trichopoulou, Pagona Lagiou, Dimitrios Trichopoulos, Domenico Palli, Valeria Pala, Rosario Tumino, Paolo Vineis, Salvatore Panico, Petra H. M. Peeters, Vincent K. Dik, Elisabete Weiderpass, Eiliv Lund, Jose Ramon Quiros Garcia, Raul Zamora-Ros, Maria José Sánchez Pérez, Miren Dorronsoro, Carmen Navarro, Eva Ardanaz, Jonas Manjer, Martin Almquist, Ingegerd Johansson, Richard Palmqvist, Kay-Tee Khaw, Nick Wareham, Timothy J. Key, Francesca L. Crowe, Veronika Fedirko, Marc J. Gunter, Elio Riboli

**Affiliations:** 1 Department of Epidemiology and Biostatistics, School of Public Health, Imperial College London, London, United Kingdom; 2 International Agency for Research on Cancer, Lyon, France; 3 The National Institute for Public Health and the Environment, Bilthoven, The Netherlands; 4 Department of Gastroenterology and Hepatology, University Medical Centre, Utrecht, The Netherlands; 5 Institute of Community Medicine, University of Tromsø, Tromsø, Norway; 6 Danish Cancer Society, Institute of Cancer Epidemiology, Copenhagen, Denmark; 7 Department of Cardiology, Aarhus University Hospital, Aalborg, Denmark; 8 Department of Epidemiology, School of Public Health, Aarhus University, Aarhus, Denmark; 9 Inserm, Centre for Research in Epidemiology and Population Health, Institut Gustave Roussy, Villejuif, France; 10 Paris South University, UMRS 1018, F-94805, Villejuif, France; 11 German Cancer Research Center, Division of Cancer Epidemiology, Heidelberg, Germany; 12 Department of Epidemiology, German Institute of Human Nutrition, Potsdam-Rehbrücke, Germany; 13 World Health Organization Collaborating Center for Food and Nutrition Policies, Department of Hygiene, Epidemiology and Medical Statistics, University of Athens Medical School, Athens, Greece; 14 Hellenic Health Foundation, Athens, Greece; 15 Department of Epidemiology, Harvard School of Public Health, Boston, Massachusetts, United States of America; 16 Bureau of Epidemiologic Research, Academy of Athens, Athens, Greece; 17 Molecular and Nutritional Epidemiology Unit, Cancer Research and Prevention Institute – ISPO, Florence, Italy; 18 Nutritional Epidemiology Unit, Fondazione IRCCS Istituto Nazionale Tumori, Milan, Italy; 19 Cancer Registry and Histopathology Unit, “Civile – M.P.Arezzo” Hospita, ASP Ragusa, Italy; 20 HuGeF Foundation, Torino, Italy; 21 Department of Clinical and Experimental Medicine, Federico II University, Naples, Italy; 22 Julius Centre, University Medical Centre Utrecht, Utrecht, The Netherlands; 23 Public Health Directorate, Asturias, Spain; 24 Unit of Nutrition, Environment and Cancer Catalan Institute of Oncology, Barcelona, Spain; 25 Andalusian School of Public Health, Granada, Spain; 26 CIBER Epidemiology and Public Health CIBERESP, Madrid, Spain; 27 Public Health Division of Gipuzkoa, Basque Regional Health Department and Ciberesp-Biodonostia, San Sebastian, Spain; 28 Department of Epidemiology, Murcia Regional Health Council, Murcia, Spain; 29 Sociosanitary Sciences Department, Universidad de Murcia, Murcia, Spain; 30 Navarre Public Health Institute, Pamplona, Spain; 31 Department of Plastic Surgery, Lund University, Malmo, Sweden; 32 Department of Surgery, Lund University, Malmo, Sweden; 33 Department of Odontology, Umeå University, Umeå, Sweden; 34 Department of Medical Biosciences, Pathology, Umeå University, Umeå, Sweden; 35 University of Cambridge, Cambridge, United Kingdom; 36 MRC Epidemiology Unit, Cambridge, United Kingdom; 37 Cancer Epidemiology Unit, Nuffield Department of Clinical Medicine, University of Oxford, Oxford, United Kingdom; Robert Gordon University, United Kingdom

## Abstract

**Background:**

Prospective studies have consistently reported lower colorectal cancer risks associated with higher intakes of total dairy products, total milk and dietary calcium. However, less is known about whether the inverse associations vary for individual dairy products with differing fat contents.

**Materials and Methods:**

In the European Prospective Investigation into Cancer and Nutrition (EPIC), we investigated the associations between intakes of total milk and milk subtypes (whole-fat, semi-skimmed and skimmed), yoghurt, cheese, and dietary calcium with colorectal cancer risk amongst 477,122 men and women. Dietary questionnaires were administered at baseline. Multivariable hazard ratios (HRs) and 95% confidence intervals (CIs) were estimated using Cox proportional hazards models, adjusted for relevant confounding variables.

**Results:**

During the mean 11 years of follow-up, 4,513 incident cases of colorectal cancer occurred. After multivariable adjustments, total milk consumption was inversely associated with colorectal cancer risk (HR per 200 g/day 0.93, 95% CI: 0.89–0.98). Similar inverse associations were observed for whole-fat (HR per 200 g/day 0.90, 95% CI: 0.82–0.99) and skimmed milk (HR per 200 g/day 0.90, 95% CI: 0.79–1.02) in the multivariable models. Inverse associations were observed for cheese and yoghurt in the categorical models; although in the linear models, these associations were non-significant. Dietary calcium was inversely associated with colorectal cancer risk (HR per 200 mg/day 0.95, 95% CI: 0.91–0.99); this association was limited to dairy sources of calcium only (HR per 200 mg/day 0.95, 95% CI: 0.91–0.99), with no association observed for non-dairy calcium sources (HR per 200 mg/day 1.00, 95% CI: 0.81–1.24).

**Conclusions:**

Our results strengthen the evidence for a possible protective role of dairy products on colorectal cancer risk. The inverse associations we observed did not differ by the fat content of the dairy products considered.

## Introduction

Colorectal cancer is the third most common cancer worldwide, with over 1.2 million new diagnoses estimated to have occurred in 2008 [1]. Variation in international incidence rates [2], [3] coupled with findings from migrant studies [4], [5] suggests that colorectal cancer etiology is influenced by modifiable lifestyle factors, such as diet. In the recent WCRF/AICR Continuous Update Project, alcoholic drinks and red and processed meat were judged to be “convincing” factors associated with increased colorectal cancer risk; whilst foods containing dietary fibre were similarly judged but associated with reduced risk [6]. For total dairy products, an updated meta-analysis (the WCRF Continuous Update Project) recently reported a 17% lower colorectal cancer risk per 400 g/day increased intake, [7] but indicated that evidence for individual products was lacking and/or uncertain.

Although an inverse association between consumption of total milk with colorectal cancer risk has been consistently observed, [7], [8] whether the fat content of milk offsets a potential anti-carcinogenic role is unclear. Animal models have shown that high-fat consumption results in bile acid production, which in turn promotes colorectal cancer, [9] but associations between milk subtypes, with different fat contents, and colorectal cancer have rarely been examined in prospective studies [10]. Similarly, how other high-fat dairy products, such as cheese and yoghurt, are associated with colorectal cancer risk is unclear, as mixed results have been reported from the handful of previous prospective studies. For cheese consumption, four prospective studies reported null associations [8], [11]–[13] and one study reported an inverse association [14]. For yoghurt, three cohort studies have not found any association, [8], [11], [12] but a recent analysis within the European Prospective Investigation into Cancer and Nutrition (EPIC)-Italy cohorts reported reduced risks amongst those with higher consumption, even after adjustment for calcium intake [15].

The principal anti-carcinogenic component contained within dairy products is believed to be calcium. Most, [8], [11], [12], [16], [17] but not all [18] cohort studies that have investigated calcium intake in relation to colorectal cancer have reported inverse associations. Previously within EPIC, a nested case-control study based on 1,248 colorectal cancer cases reported higher intakes of dietary calcium were associated with lower colorectal cancer risk [19]. Although, whether this association differed according to dairy and non-dairy sources of calcium was not investigated, nor was a potential non-linear relationship that has been observed in other cohorts [8], [11].

In this present analysis, we investigated how intakes of milk with different fat content (total, whole-fat, semi-skimmed, and skimmed), cheese, yoghurt, and dietary calcium (total, dairy and non-dairy sources) relate to colorectal cancer risk in the EPIC study. The EPIC is a large prospective cohort from 10 European countries with a wide range of dietary intakes. The large number of participants and colorectal cancer cases accrued provided high statistical power to investigate relationships according to individual dairy products and across cancer sub-sites.

## Methods

### Outline

EPIC is an on-going multicentre prospective cohort study designed to investigate the associations between diet, lifestyle, genetic and environmental factors and various types of cancer. A detailed description of the methods has previously been published [20], [21]. In summary, 521,448 participants (∼70% women) mostly aged 35 years or above were recruited between 1992 and 2000. Participants were recruited from 23 study centres in ten European countries: Denmark, France, Germany, Greece, Italy, the Netherlands, Norway, Spain, Sweden, and United Kingdom (UK). Participants were recruited from the general population, with the following exceptions: the French cohort were teacher health insurance programme members; the Italian and Spanish cohorts included members of blood donor associations and the general population; the Utrecht (the Netherlands) and Florence (Italy) cohorts contained participants from mammographic screening programs; the Oxford (UK) cohort included a large proportion of vegetarians, vegans, and low meat eaters; finally, only women participated in the cohorts of France, Norway, Naples (Italy) and Utrecht (the Netherlands). Written informed consent was provided by all study participants. Ethical approval for the EPIC study was obtained from the review boards of the International Agency for Research on Cancer (IARC) and local participating centres. Exclusions prior to the onset of the analyses included: participants with prevalent cancer at enrolment (n = 28,283); participants with missing dietary, lifestyle, and anthropometric data (n = 6,253); participants in the highest and lowest 1% of the distribution for the ratio between energy intake to estimated energy requirement (n = 9,600); and finally participants with extreme total dairy intakes above 2000 g/day (n = 190). Our study therefore included 477,122 participants (334,981 women and 142,141 men).

### Diet and lifestyle questionnaires

Dietary information over the previous 12 months was obtained at study baseline using validated country/centre specific dietary questionnaires. In Malmö (Sweden), a dietary questionnaire was combined with a 7-day food registration and interview. In Greece, two Italian centres, and Spain, interviewers administered the dietary questionnaires. In all other centres/countries, the questionnaires were self-administered. In Spain, France, and Ragusa (Italy) questions were structured by meals, while in other countries the structure was by food groups. Also at baseline, standardized computer-based single 24-hour dietary recalls (24-hdr) were collected from 36,994 study participants. This additional dietary assessment was used to calibrate for differences in questionnaires across countries [22]. Individual dairy products were categorized as milk, cheeses, and yoghurts. Due to relatively low intakes and incomplete measurements across centres, other individual dairy products such as ice cream, cream desserts and milk-based puddings, milk beverages, dairy creams and creamers for milk and coffee were not analysed individually. Total milk was assessed as the sum of all types of milk consumed (whole-fat, skimmed, semi skimmed, and not specified). Semi-skimmed milk was defined as milk containing 0.5–2.5% fat, and skimmed milk was defined as having <0.5% fat content. Milk subtype information was unavailable in Norway, and only partially available in Germany, Greece (both whole-fat milk only), and three Italian centres (Florence, Varese, Turin; whole-fat and semi-skimmed milks only). Cheese included all kinds of fresh, fermented, and matured cheese. Yoghurt included natural and flavoured in all cohorts, and additionally fermented milk in Sweden, Norway, and Denmark. Intakes of calcium were obtained from the EPIC Nutrient Data Base (ENDB); in which the nutritional composition of foods across the different countries has been standardized [23].

Lifestyle questionnaires were used to obtain information on education (used as a proxy for socioeconomic status), smoking status and intensity, alcohol consumption, and physical activity levels. Height and weight were measured at the baseline examination in all centres apart from part of Oxford, and all of the Norway and France sub-cohorts, where measurements were self-reported via the lifestyle questionnaire [20].

### Ascertainment of colorectal cancer incidence

Population cancer registries were used in Denmark, Italy, the Netherlands, Norway, Spain, Sweden and the United Kingdom to identify incident cancer diagnoses. In France, Germany and Greece cancer cases during follow-up were identified by a combination of methods including: health insurance records, cancer and pathology registries, and by active follow-up directly through study participants or through next-of-kin. Complete follow-up censoring dates varied amongst centres, ranging between 2005 and 2010.

Cancer incidence data were coded using the 10^th^ Revision of the International Classification of Diseases (ICD-10) and the second revision of the International Classification of Disease for Oncology (ICDO-2). Proximal colon cancer included those within the caecum, appendix, ascending colon, hepatic flexure, transverse colon, and splenic flexure (C18.0–18.5). Distal colon cancer included those within the descending (C18.6) and sigmoid (C18.7) colon. Overlapping (C18.8) and unspecified (C18.9) lesions of the colon were grouped among colon cancers only. Cancer of the rectum included cancer occurring at the recto sigmoid junction (C19) and rectum (C20).

### Statistical analysis

Hazard ratios (HRs) and 95% confidence intervals (CIs) were estimated using Cox proportional hazards models. Age was the primary time variable in all models. Time at entry was age at recruitment. Exit time was age at whichever of the following came first: colorectal cancer diagnosis, death, or the last date at which follow-up was considered complete in each centre. To control for differing follow-up procedures, questionnaire design, and other differences across centres, models were stratified by study centre. Models were also stratified by sex and age at recruitment in 1-year categories. Possible non-proportionality was assessed using an analysis of Schoenfeld residuals, [24] with no evidence of non-proportionality being detected.

Dietary intakes were modelled using either quintiles defined across cohort participants (total milk, total dairy and calcium); pre-defined categories (whole-fat, semi-skimmed, and skimmed milks: non consumers, <100, 100–199, 200–299, ≥300 g/day); and a predefined low intake reference category and quartiles defined across the remaining participants (cheese reference category  = <5 g/day; yoghurt reference category  =  non-consumers). Intakes were also modelled as continuous variables, with HR expressed per increments of: 200 g/day for milk; 100 g/day for yoghurt; 50 g/day for cheese; 400 g/day for total dairy intake, and 200 mg/day for calcium. Trend tests across intake categories were calculated by assigning the median value of each intake quintile/category and modelling as continuous terms into Cox regression models.

Analyses for colorectal, colon, proximal colon, distal colon, and rectal cancers were conducted for both sexes combined as no interactions by sex were observed for intakes of total dairy products (*P* = 0.26), milk (*P* = 0.28), cheese (*P* = 0.58), yoghurt (*P* = 0.51), and dietary calcium (*P* = 0.11). The results by sex are in Tables S1, S2, S3, and S4 in File S1. All models were adjusted for total energy intake, using the standard model, to obtain isocaloric risk estimates and partly control for measurement error of dairy products and calcium intake estimates. All models were additionally adjusted for: body mass index (BMI; kg/m^2^; continuous); physical activity (inactive, moderately inactive, moderately active, active, or missing); smoking status and intensity (never; current, 1–15 cigarettes per day; current, 16–25 cigarettes per day; current, 25+ cigarettes per day; former, quit ≤10 years; former, quit 11–20 years; former, quit 20+ years; current, pipe/cigar/occasional; current/former, missing; or unknown); education level (none/primary school completed, technical/professional school, secondary school, longer education – including university, or unknown); menopausal status (premenopausal, postmenopausal, perimenopausal/unknown menopausal status, or surgical postmenopausal); ever use of oral contraceptive (yes, no, or unknown); ever use of menopausal hormone therapy (yes, no, or unknown); and intakes of alcohol (yes or no; continuous, g/day), red and processed meats, and fibre (both continuous, g/day). Finer adjustment for body shape was attempted by also controlling for waist circumference in a subset of the cohort for which measurements were available. When included in the multivariable models, instead of, or with BMI, the risk estimates were virtually unchanged; and accordingly, we adjusted solely for BMI. In the analyses for whole-fat, semi-skimmed, and skimmed milk, the models included the covariates as detailed above, plus additional adjustment for the other milk subtypes. Similarly, the dairy and non-dairy calcium analyses were mutually adjusted for one another.

To determine whether the dietary calcium-colorectal cancer association differed according to anthropometric, lifestyle, and dietary characteristics, we included interaction terms (multiplicative scale) in separate models. The statistical significance of the cross-product terms were evaluated using the likelihood ratio test.

Cox proportional hazard restricted cubic spline models were used to explore possible deviation from a non-linear calcium-colorectal cancer relationship, with five knots specified at the median of each quintile of intake [25]. Heterogeneity of associations across anatomical cancer sub-sites was assessed by calculating χ^2^ statistics. The heterogeneity across countries was explored by taking a meta-analytic approach [26]. To evaluate possible reverse causality, cases diagnosed within the first 2 and 5 years of follow-up were excluded from the analyses.

To improve comparability of data across study centres and to partially correct the relative risk estimates for the measurement error of dietary intakes, a linear regression calibration model was used utilizing the 24-hdr taken at baseline from a subset of the cohort (n = 34,426 in this analysis) [27], [28]. The 24-hdr were regressed on dietary questionnaire values, with adjustment for the same list of covariates detailed above, and further control for the week day and season of recall measurements. Country and sex-specific calibration models were used to obtain individual calibrated values of dietary exposure for all participants. Cox proportional hazards regression models were then applied using the calibrated values for each participant on a continuous scale. The standard error of the de-attenuated coefficients was corrected through bootstrap sampling. The *P*-value for the trend of the de-attenuated coefficients was calculated by dividing the de-attenuated coefficient by the bootstrap-derived standard error and approximating the standardized normal distribution. (29).

Statistical tests used in the analysis were all two-sided and a *P-value* of <0.05 was considered statistically significant. Analyses were conducted using SAS version 9.1 and Stata version 11.0.

## Results

After a mean (SD) follow-up of 11.0 (2.8) years, 4,513 colorectal cancer cases were documented amongst the 477,122 participants. Of the 4,513 colorectal cancer cases, 2,868 were colon tumours (1,298 proximal; 1,266 distal and 304 overlapping or unspecified), and 1,645 were rectal tumours. The total person-years and distribution of colorectal cancer cases by country are shown in Table 1. The crude colorectal cancer incidence rates for men and women were 12 and 7 cases per 10,000 person-years respectively. Intakes of total dairy products were relatively low in Greece and Germany and higher in Spain, the Netherlands, and Sweden (men) cohorts. The lowest calcium intakes were reported in the Italian cohort, with the highest in the Netherlands, UK (men), and Germany (women). A higher proportion of current smokers were observed amongst men and women in the lowest intake quintiles of dairy products; whilst a greater proportion of physically active participants were observed amongst men and women in the highest intake quintiles (Table 2). Compared to those in the lower intake quintiles, men and women with higher reported dairy intakes tended to have lower BMIs, higher education level, and reported lower intakes of alcohol, and higher intakes of dietary fibre (Table 2).

**Table 1:** Descriptive information of the European Prospective Investigation into Cancer and Nutrition participant countries.

	Number of participants		Total person-years		Number of colorectal cancer cases		Total dairy products intake (g/day) *		Dietary calcium intake (mg/day) *	
Country	Men	Women	Men	Women	Men	Women	Men	Women	Men	Women
Denmark	26,266	28,699	284,431	316,511	474	353	246.1	215	967.8	875.8
France	–	67,372	–	699,221	–	423	–	238.5	–	841.8
Germany	21,135	27,386	208,164	271,857	263	172	143.1	167.5	867.1	879.7
Greece	10,807	15,225	99,108	148,604	61	44	142.4	142.7	933.7	762.3
Italy	14,029	30,510	158,917	341,469	173	245	160.9	180	829.6	683.8
Norway	–	35,169	–	342,279	–	210	–	215.8	–	745.7
Spain	15,147	24,849	182,950	299,557	185	144	289.4	364	946.5	886
Sweden	22,287	26,374	289,320	349,295	339	313	327.6	287	945.5	833.7
The Netherlands	9,618	26,854	115,334	315,529	81	305	281.5	342.4	1004.1	970.9
United Kingdom	22,852	52,543	252,096	586,301	324	404	288.8	288.7	1035.8	846.5
All EPIC	142,141	334,981	1,590,320	3,670,621	1,900	2,613	238.9	245.6	932.3	836.3

* Data are median intake information collected from 24-hour dietary recalls (n = 34,426 participants).

**Table 2:** Baseline characteristics of study participants by categories of total dairy intake.

Characteristic	Quintile of total dairy intake									
	Q1		Q2		Q3		Q4		Q5	
**Dairy intake range (g/day)**	<134		134–228		229–332		333–489		≥490	
**Men**										
***N***	33,251		25,578		26,580		24,183		32,549	
**Colorectal cancer cases**	484		325		343		330		418	
**Age at recruitment (years) §**	52.1	9.2	52.0	9.5	52.6	9.7	52.4	10.9	51.9	11.2
**Body mass index (kg/m2)**	26.8	3.8	26.8	3.6	26.5	3.6	26.2	3.5	26.1	3.6
**Education ‡**										
Longer education including University	24.7		26.8		26.5		28.7		26.3	
**Smoking status and intensity ‡**										
Current (%)	34.0		29.7		29.3		24.8		27.9	
**Physical activity ‡**										
Active (%)	22.7		22.4		23.9		23.5		27.8	
**Total energy intake (kcal/day)**	2233	628	2331	648	2391	641	2457	648	2629	665
**Red and processed meat intake (g/day)**	100.5	64.2	98.4	60.9	98.4	58.5	90.6	59.5	97.4	61.5
**Calcium intake (mg/day)**	685	232	871	272	991	286	1153	301	1496	398
**Fibre intake (g/day)**	23.2	8.5	23.7	8.0	24.3	8.1	24.8	8.2	25.4	8.7
**Alcohol intake (g/day)**	26.7	27.4	22.2	23.2	20.4	21.9	16.8	19.3	14.7	19.0
**Women**										
***N***	62,174		69,846		68,845		71,241		62,875	
**Colorectal cancer cases**	494		525		528		523		543	
**Age at recruitment (years) §**	50.4	9.5	50.3	9.3	50.9	9.5	50.9	10.3	51.6	10.4
**Body mass index (kg/m2)**	25.1	4.6	24.9	4.5	25	4.4	24.9	4.3	24.9	4.3
**Education ‡**										
Longer education including University	19.6		22.9		22.8		24.5		23.0	
**Smoking status and intensity ‡**										
Current (%)	24.5		20.2		18.0		16.6		18.6	
**Physical activity ‡**										
Active (%)	10.7		11.1		13.3		14.7		20.1	
**Ever use of contraceptive pill ‡**										
Yes (%)	56.1		57.7		56.4		58.0		57.9	
**Ever use of menopausal hormone therapy ‡**										
Yes (%)	22.9		24.2		24.5		24.0		25.7	
**Menopausal status ‡**										
Postmenopausal (%)	41.8		40.6		43.3		43.7		47.8	
**Total energy intake (kcal/day)**	1694	485	1834	507	1941	521	2007	527	2176	539
**Red and processed meat intake (g/day)**	62.8	42.3	65.7	41.6	66.6	41.6	64.3	42.9	66.3	44.0
**Calcium intake (mg/day)**	610	211	783	229	935	253	1094	292	1443	405
**Fibre intake (g/day)**	20.7	7.5	21.2	6.8	22.1	7.0	22.7	7.3	24.0	7.7
**Alcohol intake (g/day)**	8.7	13.2	8.3	11.9	8.2	11.6	7.4	10.6	7.0	10.4

Mean and standard deviation unless stated otherwise.

### Total milk and milk subtypes by fat content

Total milk was similarly inversely related to the cancer risk across all locations of the bowel (colon vs. rectal *P* Heterogeneity  = 0.83; distal colon vs. proximal colon *P* Heterogeneity  = 0.76) (Table 3). In calibrated models, colorectal cancer risk was 7% lower for each 200 g/day higher intake of total milk. Over 17% of participants reported consuming more than one milk subtype. The linear inverse associations for colorectal, colon, and rectal cancers were of similar strength for whole-fat and skimmed milk, but there were no significant associations for semi-skimmed milk (Table 4). However, in sensitivity analyses, when the models included only sole consumers of each milk subtype, identical inverse colorectal cancer risk estimates were observed for whole-fat (HR per 200 g/day 0.87, 95% CI: 0.79–0.95), semi-skimmed (HR per 200 g/day 0.87, 95% CI: 0.78–0.97) and skimmed milks (HR per 200 g/day 0.87, 95% CI: 0.76–0.99) (data not tabulated).

**Table 3:** Multivariable hazard ratios (95% confidence intervals) of colorectal cancer risk by dairy product consumption categories.

BOTH SEXES							Basic model	Multivariable models				
									Colon cancer			
Food group (g/day)		CRC cases (n) *			Person- years		Colorectal cancer †	Colorectal cancer ‡	All colon ‡	Proximal ‡	Distal ‡	Rectal cancer ‡
							(n = 4,513)	(n = 4,513)	(n = 2,868)	(n = 1,298)	(n = 1,266)	(n = 1,645)
**Total milk**												
Q1	<9	808			1,013,915		1.00	1.00	1.00	1.00	1.00	1.00
Q2	9–89	935			1,044,757		0.97 (0.88– 1.07)	0.97 (0.88– 1.07)	0.94 (0.83– 1.07)	0.91 (0.75– 1.10)	0.98 (0.82– 1.18)	1.02 (0.87–1.20)
Q3	90–187	836			1,037,641		0.92 (0.83– 1.02)	0.92 (0.83– 1.02)	0.95 (0.84– 1.08)	0.95 (0.78– 1.14)	0.93 (0.77– 1.13)	0.88 (0.74–1.04)
Q4	188–324	988			1,079,981		0.89 (0.80– 0.98)	0.90 (0.81– 0.99)	0.90 (0.79– 1.02)	0.88 (0.73– 1.07)	0.94 (0.77– 1.13)	0.91 (0.76–1.08)
Q5	≥325	946			1,084,647		0.80 (0.72– 0.89)	0.81 (0.73– 0.90)	0.80 (0.70–0.91)	0.84 (0.69–1.02)	0.78 (0.63– 0.96)	0.84 (0.70–0.99)
* P*-trend							<0.001	<0.001	0.001	0.11	0.009	0.017
Per 200 g/day – uncalibrated								0.94 (0.91– 0.97)	0.93 (0.90– 0.97)	0.95 (0.89– 1.01)	0.94 (0.88– 0.99)	0.95 (0.90–1.00)
Per 200 g/day – calibrated								0.93 (0.89– 0.98)	0.93 (0.88– 0.98)	0.93 (0.87– 0.99)	0.95 (0.87– 1.04)	0.94 (0.87–1.02)
**Cheese**												
	<5	495			506,354		1.00	1.00	1.00	1.00	1.00	1.00
Q1	5–18	1073			1,218,699		0.93 (0.83– 1.04)	0.92 (0.82– 1.03)	0.87 (0.76– 1.00)	0.76 (0.62– 0.92)	1.06 (0.85– 1.31)	1.01 (0.84–1.22)
Q2	19–32	1114			1,173,780		0.99 (0.88– 1.11)	0.99 (0.88– 1.11)	0.94 (0.81– 1.08)	0.84 (0.68– 1.03)	1.02 (0.81– 1.27)	1.10 (0.91–1.34)
Q3	33–55	980			1,185,851		0.88 (0.78– 0.99)	0.89 (0.79– 1.00)	0.86 (0.74– 0.99)	0.70 (0.56–0.87)	1.01 (0.80– 1.26)	0.95 (0.78–1.16)
Q4	≥56	851			1,176,256		0.86 (0.76– 0.98)	0.87 (0.76– 0.99)	0.83 (0.71– 0.97)	0.73 (0.58– 0.93)	0.91 (0.71– 1.17)	0.95 (0.76–1.18)
* P*–trend							0.009	0.02	0.047	0.054	0.2	0.23
Per 50 g/day – uncalibrated								0.95 (0.90– 1.00)	0.94 (0.88– 1.01)	0.90 (0.81– 1.01)	0.95 (0.85– 1.06)	0.96 (0.87–1.05)
Per 50 g/day – calibrated								0.92 (0.80– 1.06)	0.88 (0.75– 1.04)	0.85 (0.68– 1.05)	0.82 (0.66– 1.03)	1.00 (0.79–1.26)
**Yoghurt**												
	0	1074			1,060,510		1.00	1.00	1.00	1.00	1.00	1.00
Q1	<17.8	958			1,051,433		0.96 (0.87– 1.07)	0.97 (0.88– 1.07)	0.98 (0.86– 1.11)	1.02 (0.84– 1.23)	0.93 (0.77– 1.12)	0.96 (0.81–1.13)
Q2	17.9–53	768			1,031,493		0.91 (0.82– 1.00)	0.93 (0.84– 1.03)	0.98 (0.86– 1.11)	0.96 (0.80– 1.17)	0.97 (0.80– 1.17)	0.86 (0.72–1.02)
Q3	54–108	824			1,047,993		0.88 (0.80– 0.98)	0.92 (0.83– 1.02)	0.98 (0.86– 1.10)	1.00 (0.83– 1.20)	0.95 (0.79– 1.14)	0.82 (0.69–0.97)
Q4	≥109	889			1,069,512		0.86 (0.78– 0.95)	0.90 (0.81– 0.99)	0.88 (0.77– 1.00)	0.94 (0.79– 1.13)	0.84 (0.69– 1.02)	0.93 (0.79–1.10)
* P*–trend							0.002	0.043	0.037	0.44	0.09	0.55
Per 100 g/day – calibrated								0.99 (0.95– 1.03)	0.99 (0.94– 1.04)	1.00 (0.94– 1.07)	0.98 (0.91– 1.05)	0.99 (0.93–1.05)
Per 100 g/day – uncalibrated								0.97 (0.90– 1.04)	0.98 (0.90– 1.06)	1.02 (0.92– 1.15)	0.93 (0.82– 1.06)	0.96 (0.85–1.09)
**Total dairy**												
Q1	<134	978		1,028,047			1.00	1.00	1.00	1.00	1.00	1.00
Q2	134–228	850		1,031,665			0.89 (0.81– 0.97)	0.90 (0.82– 0.99)	0.86 (0.77– 0.97)	0.75 (0.62– 0.90)	0.91 (0.76– 1.08)	0.97 (0.83–1.13)
Q3	229–332	871		1,053,198			0.83 (0.75– 0.91)	0.85 (0.77– 0.93)	0.86 (0.76– 0.96)	0.81 (0.68– 0.97)	0.86 (0.72– 1.02)	0.83 (0.71–0.97)
Q4	333–489	853		1,065,426			0.76 (0.69– 0.84)	0.79 (0.71– 0.87)	0.78 (0.69– 0.88)	0.81 (0.68– 0.98)	0.74 (0.61– 0.89)	0.80 (0.67–0.94)
Q5	≥490	961		1,082,605			0.75 (0.68– 0.83)	0.77 (0.70– 0.86)	0.75 (0.66– 0.86)	0.75 (0.62– 0.91)	0.74 (0.61– 0.90)	0.81 (0.69–0.96)
* P*-trend							<0.001	<0.001	<0.001	0.04	0.001	0.008
Per 400 g/day – uncalibrated								0.88 (0.83– 0.93)	0.87 (0.80– 0.93)	0.90 (0.81– 1.01)	0.86 (0.77– 0.96)	0.90 (0.82–0.99)
Per 400 g/day – calibrated								0.86 (0.79– 0.94)	0.85 (0.76– 0.95)	0.89 (0.78– 1.03)	0.85 (0.73– 0.99)	0.88 (0.76–1.02)

† Basic model – Cox regression using total energy intake (continuous), and stratified by age (1-year categories), sex, and centre.

‡ Multivariable model – Cox regression using total energy intake (continuous), body mass index (continuous), physical activity index (inactive, moderately inactive, moderately active, active, or missing), smoking status and intensity (never; current, 1–15 cigarettes per day; current, 16–25 cigarettes per day; current, 16+ cigarettes per day; former, quit ≤10 years; former, quit 11–20 years; former, quit 20+ years; current, pipe/cigar/occasional; current/former, missing; unknown), education status (none, primary school completed, technical/professional school, secondary school, longer education including university, or not specified), ever use of contraceptive pill (yes, no, or unknown), ever use of menopausal hormone therapy (yes, no, or unknown), menopausal status (premenopausal, postmenopausal, perimenopausal/unknown menopausal status, or surgical postmenopausal), alcohol consumption (yes or no; and continuous) and intakes of red and processed meat and fibre (both continuous), and stratified by age (1-year categories), sex, and centre.

* Total number of colorectal cancer cases across intake categories.

**Table 4:** Multivariable hazard ratios (95% confidence intervals) of colorectal cancer risk by milk subtype by fat content consumption categories.

BOTH SEXES			Basic model	Multivariable model	Multivariable models			
					Colon cancer			
Milk subtype (g/day)	CRC cases (n) *	Person-years	Colorectal cancer †	Colorectal cancer ‡	All colon ‡	Proximal ‡	Distal ‡	Rectal cancer ‡
**Whole-fat milk φ**								
0	1397	1,624,243	1.00	1.00	1.00	1.00	1.00	1.00
<100	2256	2,530,622	1.02 (0.92–1.13)	1.03 (0.93–1.13)	1.03 (0.91–1.17)	1.09 (0.90–1.31)	1.05 (0.87–1.26)	1.02 (0.86–1.21)
100–199	259	326,905	1.00 (0.87–1.16)	0.98 (0.85–1.13)	1.02 (0.85–1.22)	1.16 (0.89–1.52)	0.98 (0.75–1.28)	0.91 (0.71–1.18)
200–299	196	219,597	0.96 (0.81–1.12)	0.92 (0.78–1.08)	0.93 (0.76–1.14)	0.97 (0.71–1.32)	0.88 (0.65–1.21)	0.89 (0.67–1.18)
≥300	195	217,295	0.90 (0.77–1.06)	0.86 (0.72–1.02)	0.83 (0.67–1.03)	0.92 (0.68–1.26)	0.82 (0.59–1.14)	0.90 (0.68–1.20)
* P*-trend			0.11	0.02	0.048	0.42	0.10	0.21
Per 200 g/day – uncalibrated				0.93 (0.88–0.98)	0.92 (0.86–0.99)	0.94 (0.85–1.04)	0.92 (0.82–1.02)	0.94 (0.85–1.03)
Per 200 g/day – calibrated				0.90 (0.82–0.99)	0.91 (0.82–1.02)	0.90 (0.79–1.03)	0.95 (0.81–1.11)	0.89 (0.76–1.04)
**Semi-skimmed milk** ¶								
0	1611	1,915,967	1.00	1.00	1.00	1.00	1.00	1.00
<100	1015	999,256	0.94 (0.84–1.04)	0.92 (0.82–1.02)	0.88 (0.77–1.01)	0.93 (0.76–1.14)	0.86 (0.70–1.05)	0.97 (0.81–1.16)
100–199	396	478,349	0.98 (0.87–1.10)	0.93 (0.82–1.05)	0.94 (0.81–1.09)	0.94 (0.75–1.17)	0.88 (0.70–1.10)	0.91 (0.74–1.12)
200–299	375	390,635	0.98 (0.87–1.11)	0.92 (0.81–1.05)	0.92 (0.79–1.07)	0.90 (0.71–1.13)	0.95 (0.76–1.20)	0.93 (0.76–1.15)
≥300	366	406,723	0.92 (0.82–1.04)	0.85 (0.75–0.97)	0.84 (0.71–0.99)	0.97 (0.77–1.22)	0.73 (0.57–0.95)	0.87 (0.70–1.08)
* P*-trend			0.44	0.042	0.13	0.80	0.10	0.18
Per 200 g/day – uncalibrated				0.96 (0.92–1.01)	0.96 (0.91–1.01)	0.98 (0.90–1.06)	0.95 (0.87–1.03)	0.97 (0.91–1.04)
Per 200 g/day – calibrated				0.97 (0.91–1.05)	0.97 (0.90–1.05)	0.99 (0.90–1.10)	0.97 (0.87–1.09)	0.98 (0.88–1.10)
**Skimmed milk** §								
0	2290	2,548,660	1.00	1.00	1.00	1.00	1.00	1.00
<100	559	616,032	1.01 (0.90–1.13)	1.01 (0.90–1.14)	1.04 (0.90–1.20)	1.08 (0.87–1.34)	1.04 (0.84–1.29)	0.97 (0.80–1.18)
100–199	232	246,904	1.05 (0.91–1.20)	1.02 (0.89–1.18)	1.03 (0.86–1.22)	0.97 (0.74–1.27)	1.09 (0.84–1.41)	1.02 (0.81–1.28)
200–299	113	149,954	0.81 (0.67–0.98)	0.78 (0.64–0.95)	0.84 (0.66–1.06)	0.99 (0.72–1.37)	0.71 (0.47–1.05)	0.67 (0.47–0.96)
≥300	216	250,916	0.81 (0.70–0.93)	0.78 (0.67–0.90)	0.72 (0.60–0.88)	0.68 (0.51–0.90)	0.79 (0.59–1.05)	0.87 (0.69–1.10)
* P*-trend			0.002	<0.001	<0.001	0.012	0.06	0.10
Per 200 g/day – uncalibrated				0.92 (0.87–0.97)	0.91 (0.85–0.97)	0.89 (0.81–0.98)	0.94 (0.85–1.04)	0.93 (0.86–1.01)
Per 200 g/day – calibrated				0.90 (0.79–1.02)	0.88 (0.78–0.99)	0.82 (0.72–0.94)	0.95 (0.83–1.10)	0.92 (0.81–1.06)

† Basic model – Cox regression using total energy intake (continuous), and stratified by age (1-year categories), sex, and centre.

‡ Multivariable model – Cox regression using total energy intake (continuous), body mass index (continuous), physical activity index (inactive, moderately inactive, moderately active, active, or missing), smoking status and intensity (never; current, 1–15 cigarettes per day; current, 16–25 cigarettes per day; current, 16+ cigarettes per day; former, quit ≤10 years; former, quit 11–20 years; former, quit 20+ years; current, pipe/cigar/occasional; current/former, missing; unknown), education status (none, primary school completed, technical/professional school, secondary school, longer education including university, or not specified), ever use of contraceptive pill (yes, no, or unknown), ever use of menopausal hormone therapy (yes, no, or unknown), menopausal status (premenopausal, postmenopausal, perimenopausal/unknown menopausal status, or surgical postmenopausal), alcohol consumption (yes or no; and continuous) and intakes of red and processed meat and fibre (both continuous), and stratified by age (1-year categories), sex, and centre.

φ Excluding Norway.

¶ Excluding Norway, Germany, and Greece.

§ Excluding Norway, Germany, Greece, Florence (Italy), Varese (Italy), and Turin (Italy).

* Total number of colorectal cancer cases across intake categories.

### Cheese

Cheese consumption was inversely associated with colorectal cancer in the categorical model (Table 3). The association was significant for colon (≥56 g/day vs. <5 g/day HR, 0.83, 95% CI: 0.71–0.97; *P*-trend  = 0.047) but not rectal cancer, although this difference was not significant (*P* Heterogeneity  = 0.39). In the linear calibrated models, non-significant inverse associations were observed for colorectal, colon and rectal cancers. For proximal colon cancer, the highest consumers (>56 g/day) had a 27% (95% CI: 0.58–0.93) reduced risk compared to those consuming <5 g/day, but in the calibrated model, this association was not significant. No association was observed for tumours in the distal region of the colon, and the heterogeneity in association by colonic region was not statistically significant (*P* Heterogeneity  = 0.82).

### Yoghurt

Yoghurt intake was significantly inversely related to colorectal cancer risk in categorical models (≥109 g/day vs. non-consumers, HR 0.90, 95% CI: 0.81–0.99; *P*-trend  = 0.043) (Table 3). The inverse association was restricted to the colon and not observed for tumours in the rectum, although the difference was not statistically significant (*P* Heterogeneity  = 0.79). Within the colon the difference in association across the distal and proximal regions was non-significant (*P* Heterogeneity  = 0.29). No associations were observed in the linear calibrated models for cancers across all bowel locations. After adjustment for dietary calcium intake the inverse association for colorectal cancer using the categorical model was no longer significant (≥109 g/day vs. non-consumers, HR 0.94, 95% CI: 0.85–1.04; *P*-trend  = 0.33; data not tabulated).

### Total dairy intake

Total dairy intake was significantly inversely associated to colorectal cancer risk (≥490 g/day vs. <134 g/day, HR 0.77, 95% CI: 0.70–0.86; *P*-trend <0.001) (Table 3). In calibrated models, each 400 g/day higher intake of total dairy products was associated with a 14% lower risk. The inverse association was of similar magnitude for colon and rectal cancer (*P* Heterogeneity  = .72); and within the colon, there no evidence of heterogeneity across distal and proximal regions (*P* Heterogeneity  = 0.66).

### Dietary calcium

For dietary calcium, similar strength inverse associations were observed across all locations of the colorectum (colon vs. rectal *P* Heterogeneity  = 0.56; distal colon vs. proximal colon *P* Heterogeneity  = 1.00) (Table 5). There was no deviation from linearity for the relationship between dietary calcium and colorectal cancer in the restricted cubic spline model (*P* = 0.43) (data not shown). Calcium intake from dairy foods was inversely associated to cancer risk across all locations of the bowel. When calcium and milk were included in the same models, the inverse associations for milk weakened and became non-significant, but the significant inverse associations for calcium remained (data not shown). Dietary calcium from non-dairy sources was not inversely associated with colorectal cancer risk. The association between dietary calcium intake and risk of colorectal cancer did not differ by BMI (*P* = 0.56), waist circumference (men *P* = 0.74; women *P* = 0.64), physical activity (*P* = 0.26), smoking status (*P* = 0.37 alcohol consumption (*P* = 0.75), and intakes of red and processed meat (*P* = 0.50), and fibre (*P* = 0.65) (data not tabulated).

**Table 5:** Multivariable hazard ratios (95% confidence intervals) of colorectal cancer risk by dietary calcium intake categories.

BOTH SEXES				Basic model	Multivariable model	Multivariable models					
						Colon cancer					
		CRC cases (n) *	Person-years	Colorectal cancer †	Colorectal cancer ‡	All colon ‡		Proximal ‡		Distal ‡	Rectal cancer ‡
				(n = 4,513)	(n = 4,513)	(n = 2,868)		(n = 1,298)		(n = 1,266)	(n = 1,645)
**Calcium (mg/day)**											
Q1	<661	943	1,034,125	1.00	1.00	1.00	1.00		1.00		1.00
Q2	662–845	885	1,048,337	0.87 (0.79– 0.95)	0.88 (0.80–0.97)	0.92 (0.82–1.04)	0.94 (0.79–1.12)		0.89 (0.74–1.06)		0.82 (0.70–0.96)
Q3	846– 1030	921	1,054,640	0.86 (0.78– 0.94)	0.89 (0.81–0.98)	0.90 (0.80–1.02)	0.85 (0.71–1.03)		0.96 (0.80–1.15)		0.87 (0.74–1.02)
Q4	1031–1279	891	1,059,887	0.78 (0.70– 0.87)	0.82 (0.74–0.91)	0.81 (0.71–0.93)	0.80 (0.66–0.98)		0.76 (0.63–0.93)		0.83 (0.70–0.98)
Q5	≥1280	873	1,063,951	0.73 (0.65– 0.82)	0.78 (0.69–0.88)	0.75 (0.65–0.88)	0.77 (0.62–0.97)		0.72 (0.58–0.91)		0.82 (0.67–0.99)
* P*-trend				<0.001	<0.001	<0.001	0.014		0.003		0.12
Per 200 mg/day – uncalibrated					0.95 (0.93–0.97)	0.95 (0.93–0.98)	0.95 (0.91–0.99)		0.96 (0.92–0.99)		0.96 (0.92–0.99)
Per 200 mg/day – calibrated					0.95 (0.91–0.99)	0.94 (0.90–0.98)	0.94 (0.89–0.99)		0.94 (0.88–0.99)		0.96 (0.91-1.02)
**Dairy calcium** **(mg/day)**											
Q1	<308	998	1,027,736	1.00	1.00	1.00	1.00		1.00		1.00
Q2	309–462	869	1,038,754	0.84 (0.77– 0.92)	0.85 (0.78–0.94)	0.86 (0.76–0.96)	0.75 (0.63–0.90)		0.99 (0.83–1.17)		0.85 (0.73–0.99)
Q3	463–621	866	1,049,098	0.81 (0.74– 0.89)	0.83 (0.76–0.91)	0.84 (0.74–0.94)	0.85 (0.72–1.02)		0.83 (0.69–0.99)		0.82 (0.70–0.97)
Q4	622–838	866	1,061,151	0.75 (0.68– 0.83)	0.78 (0.70–0.86)	0.78 (0.69–0.88)	0.76 (0.63–0.91)		0.78 (0.65–0.94)		0.78 (0.66–0.92)
Q5	≥839	909	1,076,084	0.75 (0.68– 0.83)	0.78 (0.70–0.87)	0.75 (0.66–0.86)	0.73 (0.60–0.89)		0.78 (0.63–0.95)		0.83 (0.70–0.99)
* P*–trend				<0.001	<0.001	<0.001	0.009		0.003		0.053
Per 200 mg/day – uncalibrated					0.95 (0.93–0.97)	0.95 (0.92–0.97)	0.95 (0.91–0.98)		0.95 (0.92–0.99)		0.96 (0.92–0.99)
Per 200 mg/day – calibrated					0.95 (0.91–0.99)	0.94 (0.90–0.99)	0.94 (0.89–1.00)		0.94 (0.89–1.00)		0.96 (0.90–1.03)
**Non–dairy calcium (mg/day)**											
Q1	<276	816	1,060,798	1.00	1.00	1.00	1.00		1.00		1.00
Q2	277–344	971	1,061,241	1.07 (0.97– 1.19)	1.10 (0.99-1.22)	1.10 (0.96–1.25)	1.07 (0.88–1.31)		1.13 (0.93–1.37)		1.11 (0.93–1.32)
Q3	345–410	975	1,054,658	1.05 (0.94– 1.17)	1.09 (0.97–1.23)	1.12 (0.97–1.29)	1.25 (1.01–1.55)		1.03 (0.82–1.28)		1.05 (0.87–1.27)
Q4	411–501	891	1,044,603	0.96 (0.85– 1.09)	1.01 (0.89–1.16)	1.05 (0.89–1.23)	1.04 (0.81–1.33)		1.08 (0.85–1.38)		0.96 (0.77–1.20)
Q5	≥502	855	1,031,522	0.98 (0.85– 1.12)	1.05 (0.90–1.23)	1.09 (0.89–1.32)	1.18 (0.88–1.59)		0.98 (0.72–1.32)		0.99 (0.76–1.29)
* P*–trend				0.244	0.87	0.77	0.45		0.64		0.52
Per 200 mg/day – uncalibrated					1.02 (0.94–1.10)	1.06 (0.96–1.17)	1.05 (0.91–1.21)		1.07 (0.92–1.25)		0.95 (0.83–1.08)
Per 200 mg/day – calibrated					1.00 (0.81–1.24)	1.06 (0.87–1.30)	0.94 (0.76–1.15)		1.15 (0.92–1.43)		0.92 (0.73–1.15)

† Basic model – Cox regression using total energy intake (continuous), and stratified by age (1-year categories), sex, and centre.

‡ Multivariable model – Cox regression using total energy intake (continuous), body mass index (continuous), physical activity index (inactive, moderately inactive, moderately active, active, or missing), smoking status and intensity (never; current, 1–15 cigarettes per day; current, 16–25 cigarettes per day; current, 16+ cigarettes per day; former, quit ≤10 years; former, quit 11–20 years; former, quit 20+ years; current, pipe/cigar/occasional; current/former, missing; unknown), education status (none, primary school completed, technical/professional school, secondary school, longer education including university, or not specified), ever use of contraceptive pill (yes, no, or unknown), ever use of menopausal hormone therapy (yes, no, or unknown), menopausal status (premenopausal, postmenopausal, perimenopausal/unknown menopausal status, or surgical postmenopausal), alcohol consumption (yes or no; and continuous) and intakes of red and processed meat, fibre, and mutual adjustment for other dietary calcium source (all continuous), and stratified by age (1-year categories), sex, and centre.

* Total number of colorectal cancer cases across intake categories.

### Between country heterogeneity and inclusion of preclinical disease

There was evidence of significant heterogeneity by country for total dairy products (*P* = 0.034) (Figure S1 in File S1); although risk estimates ≤1 were observed in most countries. No associations were observed in the Sweden and Denmark cohorts. Non-significant between country heterogeneity was observed for intakes of dietary calcium (*P* = 0.60; Figure S2 in File S1), total milk (*P* = 0.13), cheese (*P* = 0.64), and yoghurt (*P* = 0.12).

Excluding the participants with less than 2 and 5 years of follow-up (including 502 and 1,483 colorectal cancer cases respectively) from the total dairy, total milk, cheese, yoghurt, and calcium intake analyses resulted in negligible differences in the colorectal cancer associations (data not shown).

## Discussion

In this analysis of the EPIC cohort, after a mean follow-up of 11 years where 4,513 cases accrued, higher intakes of all subtypes of milk, cheese, yoghurt, total dairy products and dietary calcium from dairy sources were associated with reduced colorectal cancer risk. Overall, our results provided no evidence for divergent relationships for high and low-fat dairy products with colorectal cancer risk.

The inverse association we observed for total milk consumption was similar to what was reported by both the Pooling Project of cohort studies, and a recent systematic review [7], [8]. Few prospective studies have previously investigated the associations for milk by fat content. In the Adventist Health Study, a stronger inverse association was reported for non-fat milk consumers compared to consumers of milks containing higher fat [10]. In our larger analysis, similar strength inverse associations were observed for all milk subtypes, refuting the notion that the milk-colorectal cancer association differs according to fat content, at least in the range of intakes recorded within EPIC.

The inverse cheese-colorectal cancer association observed in the categorical models provides further evidence that the fat content of dairy products does not impair any possible anti-carcinogenic role. However, this inverse association was not replicated in the linear calibrated model, where a non-significant lower risk was yielded. For cheese consumption, results from the limited previous research have usually reported null results [11]–[13]. For yoghurt, an inverse colorectal cancer association in the categorical model was also not replicated in the linear calibrated model. Some evidence suggests that lactic acid bacteria contained within yoghurt products may protect against colorectal cancer [29]. Recently, an analysis of the EPIC-Italy cohorts reported a 35% reduced colorectal cancer risk - after adjustment for calcium intake – amongst participants who consumed more than 25 g/day of yoghurt compared to non-consumption (less than 1 g/day) [15]. When we additionally adjusted for calcium intake, the inverse colorectal association in the categorical model disappeared. However, our results do not rule out the lactic acid hypothesis, as the types of yoghurt consumed across EPIC countries may differ in lactic acid content, and this information may not have been captured within our study.

We observed consistent inverse associations across all cancer sub-sites for dietary calcium intake, in line with the majority of published cohort studies [8], [11], [12], [16], [17]. Some studies have reported a threshold level for calcium intake (∼1,000 to ∼1,400 mg/day), above which reductions in colorectal cancer risk are not observed [8], [11]. In our analysis we did not observe a threshold association at any level of intake, or any departure from linearity. Our inverse associations were limited to dairy sources of calcium, as we observed either null or weak non-statistically significant associations in the non-dairy calcium models. Other prospective studies have reported no association [18], [30] or increased risk [16] for non-dairy calcium intake with colorectal and colon cancers. A possible explanation for the non-inverse associations for non-dairy calcium could be that plant sources of calcium – the main contributors to non-dairy calcium intake amongst EPIC participants –contain oxalate and phytate which have been shown to inhibit calcium absorption [31]. Across all EPIC centres, milk contributes most to the consumption of total dairy products [32]. Lactose and casein in milk may increase the bioavailability of calcium, which could also explain the inverse associations we observed for dairy calcium [33]. The primary anti-carcinogenic component contained within dairy foods is believed to be calcium [29]. Laboratory studies have shown that calcium can induce apoptosis in colonic epithelium cells, [34] and alter colonic K-*ras* gene mutations [35]. Animal and human intervention studies have shown that calcium impacts upon colonic cell differentiation: indirectly, by binding to available bile acids and fatty acids, suppressing their ability to modify colonic cell proliferation [36], [37]; and directly, by suppressing colonic epithelial cell proliferation and inducing terminal differentiation [38]. Evidence from clinical trials suggests that calcium supplementation reduces the recurrence of colorectal adenoma [39]. Beyond the calcium content of dairy products, other constituents contained within these products may explain the inverse associations observed. For instance lactoferrin, vitamin D in fortified dairy products, and certain fatty acids, such as butyric acid, have been linked with having possible beneficial roles against colorectal cancer. [29] However, isolating the influence of individual food components present simultaneously in the same foods is difficult.

The public health implications of our results are complicated by the contrasting associations between calcium intake and prostate cancer. Dietary calcium has been consistently associated with increased prostate cancer risk, and the WCRF/AICR 2007 Expert report judged it a “probable” cause of the disease [40]. Within EPIC, a 300 mg/day intake of dietary calcium was previously associated with a 9% increased risk of prostate cancer [41]. In our analysis, an equivalent daily intake amongst men and women would be associated with 7% statistically significant reduced colorectal cancer risks. At present, the available evidence for the divergent associations between cancer sites has not been considered convincing enough to justify potential sex-specific calcium and dairy product intake recommendations.

Strengths of our study include its large-scale prospective design, the large number of colorectal cancer cases and the possibility of controlling for the main potential confounders. However, information on past bowel cancer screening and previous endoscopy procedures were unknown; although previous studies have observed unchanged inverse calcium-colon cancer relationships when the multivariable models were additionally adjusted for endoscopy history [30]. A further limitation was that intake of calcium supplements could not be included in our analysis; although other large cohort studies have observed only minor differences in associations between total calcium intakes (supplements plus diet) compared solely to dietary sources [8], [17]. In our study, diet was assessed through dietary questionnaires, which are subject to measurement error. Random misclassification may have thus caused an attenuation of the estimates of the diet-disease association; however, we partially corrected our estimates through regression calibration using 24-hdr data [28]. Another study limitation was that changes in diet during follow-up could not be taken into account; however, this does not appear to have influenced our conclusions since our results are consistent with those of other cohort studies, some of which used cumulative estimates of diet over time [8], [30].

In conclusion, our study supports potential beneficial roles for dietary intakes of dairy products and calcium on colorectal cancer prevention. Inverse associations were observed for low-fat and high-fat dairy products; indicating that the fat content contained within dairy products does not influence this relationship.

### Ethical approval review board information for local EPIC centres

The National Committee on Health Research Ethics (Denmark); Comité de Protection des Personnes (France); Ethics Committee of the Heidelberg University Medical School (Heidelberg, Germany); Ethikkommission der Landesärztekammer Brandenburg Cottbus (Potsdam, Germany); University of Athens Medical School (Greece) Comitato Etico Indipendente, Fondazione IRCCS Istituto Nazionale dei Tumori, Milano (Italy); Human Genetics Foundation Torino: Ethics Committee (Turn, Italy); The Medical Ethical Committee (METC =  Medisch Ethische Toetsingscommissie) of the University Medical Center Utrecht (UMCU), Utrecht, the Netherlands (The Netherlands); Regional ethical committee for Northern Norway and the Norwegian Data Inspectorate (Norway); CEIC Comité de Ética de Investigación Clínica (Spain); Ethics Committee of Lundst University (Malmö, Sweden); Umea Regional Ethical Review Board (Umea, Sweden); Norwich District Ethics Committee (Cambridge, UK); Scotland A Research Ethics Committee (Oxford); and the Imperial College Research Ethics Committee [ICREC] (UK).

## Supporting Information

File S1 **Supporting information.** Figure S1. Multivariable hazard ratios and 95% confidence intervals of colorectal cancer risk by country, per 400 g/day increase in total dairy intake. Hazard ratios estimated by Cox proportional hazards models adjusting for total energy intake (continuous), body mass index (continuous), physical activity index (inactive, moderately inactive, moderately active, active, or missing), smoking status and intensity (never; current, 1–15 cigarettes per day; current, 16–25 cigarettes per day; current, 16+ cigarettes per day; former, quit ≤10 years; former, quit 11–20 years; former, quit 20+ years; current, pipe/cigar/occasional; current/former, missing; unknown), education status (none, primary school completed, technical/professional school, secondary school, longer education including university, or not specified), ever use of contraceptive pill (yes, no, or unknown), ever use of menopausal hormone therapy (yes, no, or unknown), menopausal status (premenopausal, postmenopausal, perimenopausal/unknown menopausal status, or surgical postmenopausal), alcohol consumption (yes or no; and continuous) and intakes of red and processed meat and fibre (both continuous), and stratified by age (1-year categories), sex, and centre. Figure S2. Multivariable hazard ratios and 95% confidence intervals of colorectal cancer risk by country, per 200 mg/day increase in total dietary calcium (B). Hazard ratios estimated by Cox proportional hazards models adjusting for total energy intake (continuous), body mass index (continuous), physical activity index (inactive, moderately inactive, moderately active, active, or missing), smoking status and intensity (never; current, 1–15 cigarettes per day; current, 16–25 cigarettes per day; current, 16+ cigarettes per day; former, quit ≤10 years; former, quit 11–20 years; former, quit 20+ years; current, pipe/cigar/occasional; current/former, missing; unknown), education status (none, primary school completed, technical/professional school, secondary school, longer education including university, or not specified), ever use of contraceptive pill (yes, no, or unknown), ever use of menopausal hormone therapy (yes, no, or unknown), menopausal status (premenopausal, postmenopausal, perimenopausal/unknown menopausal status, or surgical postmenopausal), alcohol consumption (yes or no; and continuous) and intakes of red and processed meat and fibre (both continuous), and stratified by age (1-year categories), sex, and centre. Table S1. Multivariable hazard ratios (95% confidence intervals) of colorectal cancer risk in men by dairy product consumption categories. Table S2. Multivariable hazard ratios (95% confidence intervals) of colorectal cancer risk in women by dairy product consumption categories. Table S3. Multivariable hazard ratios (95% confidence intervals) of colorectal cancer risk in men by dietary calcium intake categories. Table S4. Multivariable hazard ratios (95% confidence intervals) of colorectal cancer risk in women by dietary calcium intake categories.
(DOCX)

## References

[1] Ferlay J, Shin HR, Bray F, Forman D, Mathers C, et al (2010) Estimates of worldwide burden of cancer in 2008: GLOBOCAN 2008. Int J Cancer 127: 2893–2917 10.1002/ijc.25516. 21351269 10.1002/ijc.25516

[2] Parkin DM, Bray F, Ferlay J, Pisani P (2005) Global Cancer Statistics, 2002. CA: A Cancer Journal for Clinicians 55: 74–108 10.3322/canjclin.55.2.74. 15761078 10.3322/canjclin.55.2.74

[3] Kamangar F, Dores GM, Anderson WF (2006) Patterns of Cancer Incidence, Mortality, and Prevalence Across Five Continents: Defining Priorities to Reduce Cancer Disparities in Different Geographic Regions of the World. Journal of Clinical Oncology 24: 2137–2150. 16682732 10.1200/JCO.2005.05.2308

[4] McMichael AJ, McCall MG, Hartshore JM, Woodings TL (1980) Patterns of gastro-intestinal cancer in european migrants to Australia: The role of dietary change. Int J Cancer 25: 431–437 10.1002/ijc.2910250402. 7372370 10.1002/ijc.2910250402

[5] Haenszel W, Kurihara M (1968) Studies of Japanese migrants. I. Mortality from cancer and other diseases among Japanese in the United States. J Natl Cancer Inst 40: 43–68 doi:10.1371/journal.pmed.0040325 5635018

[6] World Cancer Research Fund/American Institute for Cancer Research (2011) Continuous Update Project Report Summary. Food, Nutrition, Physical Activity, and the Prevention of Colorectal Cancer.

[7] Aune D, Lau R, Chan DSM, Vieira R, Greenwood DC, et al.. (2011) Dairy products and colorectal cancer risk: a systematic review and meta-analysis of cohort studies. Annals of Oncology . 10.1093/annonc/mdr269 21617020

[8] Cho E, Smith-Warner SA, Spiegelman D, Beeson WL, van den Brandt PA, et al (2004) Dairy Foods, Calcium, and Colorectal Cancer: A Pooled Analysis of 10 Cohort Studies. Journal of the National Cancer Institute 96: 1015–1022. 15240785 10.1093/jnci/djh185

[9] Narisawa T, Reddy B, Weisburger J (1978) Effect of bile acids and dietary fat on large bowel carcinogenesis in animal models. Journal of Gastroenterology 13: 206–212. 10.1007/BF02773665 689337

[10] Singh PN, Fraser GE (1998) Dietary Risk Factors for Colon Cancer in a Low-risk Population. American Journal of Epidemiology 148: 761–774. 9786231 10.1093/oxfordjournals.aje.a009697

[11] Larsson SC, Bergkvist L, Rutegård J, Giovannucci E, Wolk A (2006) Calcium and dairy food intakes are inversely associated with colorectal cancer risk in the Cohort of Swedish Men. The American Journal of Clinical Nutrition 83: 667–673. 16522915 10.1093/ajcn.83.3.667

[12] Kesse E, Boutron-Ruault MC, Norat T, Riboli E, Clavel-Chapelon F, et al (2005) Dietary calcium, phosphorus, vitamin D, dairy products and the risk of colorectal adenoma and cancer among French women of the E3N-EPIC prospective study. Int J Cancer 117: 137–144 10.1002/ijc.21148. 15880532 10.1002/ijc.21148

[13] Sanjoaquin MA, Appleby PN, Thorogood M, Mann JI, Key TJ (2004) Nutrition, lifestyle and colorectal cancer incidence: a prospective investigation of 10,998 vegetarians and non-vegetarians in the United Kingdom. Br J Cancer 90: 118–121. 14710217 10.1038/sj.bjc.6601441 PMC2395312

[14] Larsson SC, Bergkvist L, Wolk A (2005) High-fat dairy food and conjugated linoleic acid intakes in relation to colorectal cancer incidence in the Swedish Mammography Cohort. The American Journal of Clinical Nutrition 82: 894–900. 16210722 10.1093/ajcn/82.4.894

[15] Pala V, Sieri S, Berrino F, Vineis P, Sacerdote C, et al (2011) Yogurt consumption and risk of colorectal cancer in the Italian European prospective investigation into cancer and nutrition cohort. Int J Cancer 129: 2712–2719 10.1002/ijc.26193. 21607947 10.1002/ijc.26193

[16] Kampman E, Goldbohm RA, van den Brandt PA, van't Veer P (1994) Fermented Dairy Products, Calcium, and Colorectal Cancer in the Netherlands Cohort Study. Cancer Research 54: 3186–3190. 8205538

[17] Park Y, Leitzmann MF, Subar AF, Hollenbeck A, Schatzkin A (2009) Dairy Food, Calcium, and Risk of Cancer in the NIH-AARP Diet and Health Study. Arch Intern Med 169: 391–401. 19237724 10.1001/archinternmed.2008.578 PMC2796799

[18] Jarvinen R, Knekt P, Hakulinen T, Aromaa A (2001) Prospective study on milk products, calcium and cancers of the colon and rectum. Eur J Clin Nutr 55: 1000–1007. 11641750 10.1038/sj.ejcn.1601260

[19] Mazda J, Bueno-de-Mesquita HB, Pietro F, Franzel JB, Teresa N, et al.. (2010) Association between pre-diagnostic circulating vitamin D concentration and risk of colorectal cancer in European populations:a nested case-control study. BMJ 340. 10.1136/bmj.b5500 PMC2809840 20093284

[20] Riboli E, Kaaks R (1997) The EPIC Project: rationale and study design. European Prospective Investigation into Cancer and Nutrition. International Journal of Epidemiology 26: S6. 9126529 10.1093/ije/26.suppl_1.s6

[21] Riboli E, Hunt KJ, Slimani N, Ferrari P, Norat T, et al (2002) European Prospective Investigation into Cancer and Nutrition (EPIC): study populations and data collection. Public Health Nutrition 5: 1113–1124. 12639222 10.1079/PHN2002394

[22] Slimani N, Kaaks R, Ferrari P, Casagrande C, Clavel-Chapelon F, et al (2002) European Prospective Investigation into Cancer and Nutrition (EPIC) calibration study: rationale, design and population characteristics. Public Health Nutrition 5: 1125–1145. 12639223 10.1079/PHN2002395

[23] Slimani N, Deharveng G, Unwin I, Southgate DAT, Vignat J, et al (2007) The EPIC nutrient database project (ENDB): a first attempt to standardize nutrient databases across the 10 European countries participating in the EPIC study. Eur J Clin Nutr 61: 1037–1056. 17375121 10.1038/sj.ejcn.1602679

[24] Schoenfeld D (1982) Partial residuals for the proportional hazards regression model. Biometrika 69: 239–241.

[25] Durrleman S, Simon R (1989) Flexible regression models with cubic splines. Statist Med 8: 551–561 10.1002/sim.4780080504. 10.1002/sim.4780080504 2657958

[26] Greenland S, Longnecker MP (1992) Methods for Trend Estimation from Summarized Dose-Response Data, with Applications to Meta-Analysis. American Journal of Epidemiology 135: 1301–1309. 1626547 10.1093/oxfordjournals.aje.a116237

[27] Rosner B, Willett WC, Spiegelman D (1989) Correction of logistic regression relative risk estimates and confidence intervals for systematic within-person measurement error. Statist Med 8: 1051–1069 10.1002/sim.4780080905. 10.1002/sim.4780080905 2799131

[28] Ferrari P, Day NE, Boshuizen HC, Roddam A, Hoffmann K, et al (2008) The evaluation of the diet/disease relation in the EPIC study: considerations for the calibration and the disease models. International Journal of Epidemiology 37: 368–378. 18180242 10.1093/ije/dym242

[29] Norat T, Riboli E (2003) Dairy products and colorectal cancer. A review of possible mechanisms and epidemiological evidence. Eur J Clin Nutr 57: 1–17. 12548291 10.1038/sj.ejcn.1601522

[30] Wu K, Willett WC, Fuchs CS, Colditz GA, Giovannucci EL (2002) Calcium Intake and Risk of Colon Cancer in Women and Men. Journal of the National Cancer Institute 94: 437–446. 11904316 10.1093/jnci/94.6.437

[31] Weaver CM, Proulx WR, Heaney R (1999) Choices for achieving adequate dietary calcium with a vegetarian diet. The American Journal of Clinical Nutrition 70: 543S–548S. 10479229 10.1093/ajcn/70.3.543s

[32] Hjartaker A, Lagiou A, Slimani N, Lund E, Chirlaque MD, et al (2002) Consumption of dairy products in the European Prospective Investigation into Cancer and Nutrition (EPIC) cohort: data from 35955 24-hour dietary recalls in 10 European countries. Public Health Nutrition 5: 1259–1271. 12639231 10.1079/PHN2002403

[33] Gueguen L, Pointillart A (2000) The Bioavailability of Dietary Calcium. Journal of the American College of Nutrition 19: 119S–136S. 10759138 10.1080/07315724.2000.10718083

[34] Lamprecht SA, Lipkin M (2001) Cellular Mechanisms of Calcium and Vitamin D in the Inhibition of Colorectal Carcinogenesis. Annals of the New York Academy of Sciences 952: 73–87 10.1111/j.1749–6632.2001.tb02729.x. 11795445 10.1111/j.1749-6632.2001.tb02729.x

[35] Llor X, Jacoby RF, Teng BB, Davidson NO, Sitrin MD, et al (1991) K-ras Mutations in 1,2-Dimethylhydrazine-induced Colonic Tumors: Effects of Supplemental Dietary Calcium and Vitamin D Deficiency. Cancer Research 51: 4305–4309. 1868452

[36] Lapre JA, De Vries HT, Koeman JH, Van der Meer R (1993) The Antiproliferative Effect of Dietary Calcium on Colonic Epithelium Is Mediated by Luminal Surfactants and Dependent on the Type of Dietary Fat. Cancer Research 53: 784–789. 8428359

[37] Govers MJAP, Termont DSML, Lapré JA, Kleibeuker JH, Vonk RJ, et al (1996) Calcium in Milk Products Precipitates Intestinal Fatty Acids and Secondary Bile Acids and Thus Inhibits Colonic Cytotoxicity in Humans. Cancer Research 56: 3270–3275. 8764120

[38] Holt PR, Atillasoy EO, Gilman J, Guss J, Moss SF, et al (1998) Modulation of Abnormal Colonic Epithelial Cell Proliferation and Differentiation by Low-Fat Dairy Foods. JAMA: The Journal of the American Medical Association 280: 1074–1079. 9757855 10.1001/jama.280.12.1074

[39] Carroll C, Cooper K, Papaioannou D, Hind D, Pilgrim H, et al (2010) Supplemental calcium in the chemoprevention of colorectal cancer: A systematic review and meta-analysis. Clinical Therapeutics 32: 789–803 doi:10.1016/j.clinthera.2010.04.024 20685491 10.1016/j.clinthera.2010.04.024

[40] World Cancer Research Fund/American Institute for Cancer (2007) Food, Nutrition, Physical Activity, and the Prevention of Cancer: a Global Perspective.

[41] Allen NE, Key TJ, Appleby PN, Travis RC, Roddam AW, et al (2008) Animal foods, protein, calcium and prostate cancer risk: the European Prospective Investigation into Cancer and Nutrition. Br J Cancer 98: 1574–1581. 18382426 10.1038/sj.bjc.6604331 PMC2391107

