# Supplementary material for: Consumption of Dairy Products and Colorectal Cancer in the European Prospective Investigation into Cancer and Nutrition (EPIC)
Source: PLoS One. 2013 Sep 2;8(9):e72715. doi: 10.1371/journal.pone.0072715 (PMC3759377; doi:10.1371/journal.pone.0072715)
Supplement: File S1 — Supporting information. Figure S1. Multivariable hazard ratios and 95% confidence intervals of colorectal cancer risk by country, per 400 g/day increase in total dairy intake. Hazard ratios estimated by Cox proportional hazards models adjusting for total energy intake (continuous), body mass index (continuous), physical activity index (inactive, moderately inactive, moderately active, active, or missing), smoking status and intensity (never; current, 1–15 cigarettes per day; current, 16–25 cigarettes per day; current, 16+ cigarettes per day; former, quit ≤10 years; former, quit 11–20 years; former, quit 20+ years; current, pipe/cigar/occasional; current/former, missing; unknown), education status (none, primary school completed, technical/professional school, secondary school, longer education including university, or not specified), ever use of contraceptive pill (yes, no, or unknown), ever use of menopausal hormone therapy (yes, no, or unknown), menopausal status (premenopausal, postmenopausal, perimenopausal/unknown menopausal status, or surgical postmenopausal), alcohol consumption (yes or no; and continuous) and intakes of red and processed meat and fibre (both continuous), and stratified by age (1-year categories), sex, and centre. Figure S2. Multivariable hazard ratios and 95% confidence intervals of colorectal cancer risk by country, per 200 mg/day increase in total dietary calcium (B). Hazard ratios estimated by Cox proportional hazards models adjusting for total energy intake (continuous), body mass index (continuous), physical activity index (inactive, moderately inactive, moderately active, active, or missing), smoking status and intensity (never; current, 1–15 cigarettes per day; current, 16–25 cigarettes per day; current, 16+ cigarettes per day; former, quit ≤10 years; former, quit 11–20 years; former, quit 20+ years; current, pipe/cigar/occasional; current/former, missing; unknown), education status (none, primary school completed, technical/professio [file pone.0072715.s001.docx]

**Figure S1.** Multivariable hazard ratios and 95% confidence intervals of colorectal cancer risk by country, per 400 g/day increase in total dairy intake.


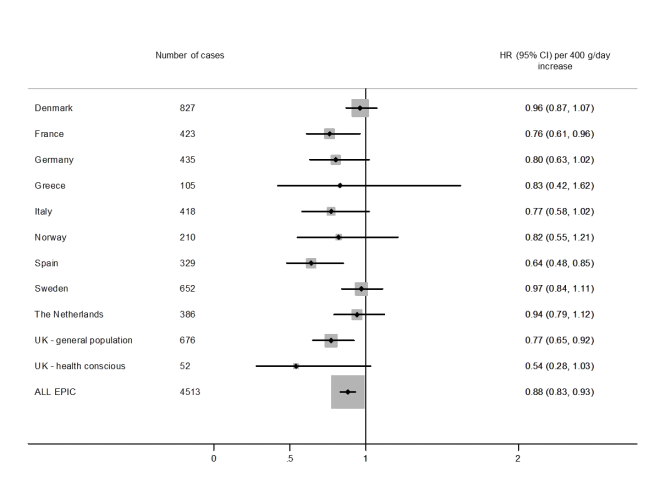


Cox regression using total energy intake (continuous), body mass index (continuous), physical activity index (inactive, moderately inactive, moderately active, active, or missing), smoking status and intensity (never; current , 1-15 cigarettes per day; current, 16-25 cigarettes per day; current, 16+ cigarettes per day; former, quit ≤10 years; former, quit 11-20 years; former, quit 20+ years; current, pipe/cigar/occasional; current/former, missing; unknown), education status (none, primary school completed, technical/professional school, secondary school, longer education including university, or not specified), ever use of contraceptive pill (yes, no, or unknown), ever use of menopausal hormone therapy (yes, no, or unknown), menopausal status (premenopausal, postmenopausal, perimenopausal/unknown menopausal status, or surgical postmenopausal), alcohol consumption (yes or no; and continuous) and intakes of red and processed meat and fibre (both continuous), and stratified by age (1-year categories), sex, and centre.

**Figure S2.** Multivariable hazard ratios and 95% confidence intervals of colorectal cancer risk by country, per 200 mg/day increase in total dietary calcium.


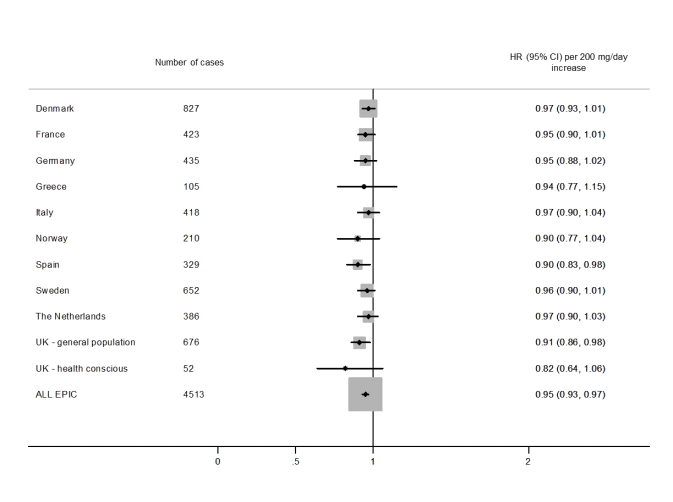


Cox regression using total energy intake (continuous), body mass index (continuous), physical activity index (inactive, moderately inactive, moderately active, active, or missing), smoking status and intensity (never; current , 1-15 cigarettes per day; current, 16-25 cigarettes per day; current, 16+ cigarettes per day; former, quit ≤10 years; former, quit 11-20 years; former, quit 20+ years; current, pipe/cigar/occasional; current/former, missing; unknown), education status (none, primary school completed, technical/professional school, secondary school, longer education including university, or not specified), ever use of contraceptive pill (yes, no, or unknown), ever use of menopausal hormone therapy (yes, no, or unknown), menopausal status (premenopausal, postmenopausal, perimenopausal/unknown menopausal status, or surgical postmenopausal), alcohol consumption (yes or no; and continuous) and intakes of red and processed meat and fibre (both continuous), and stratified by age (1-year categories), sex, and centre.

**Table S1.** Multivariable hazard ratios (95% confidence intervals) of colorectal cancer risk in men by dairy product consumption categories.

| **MEN** | |  |  | **Basic model** | **Multivariable model** | **Multivariable models** | | | |
| --- | --- | --- | --- | --- | --- | --- | --- | --- | --- |
|  |  |  |  |  |  |  | **Colon cancer** |  |  |
| **Food group (g/day)** | | | **CRC cases (n) *** | **Colorectal cancer †** | **Colorectal cancer ‡** | **All colon ‡** | **Proximal ‡** | **Distal ‡** | **Rectal cancer ‡** |
|  |  |  |  | **(n = 1,900)** | **(n = 1,900)** | **(n = 1,116)** | **(n = 478)** | **(n = 520)** | **(n = 784)** |
| **Total milk** | | |  |  |  |  |  |  |  |
|  | Q1 | <14 | 382 | 1.00 | 1.00 | 1.00 | 1.00 | 1.00 | 1.00 |
|  | Q2 | 15-99 | 383 | 0.94 (0.81-1.08) | 0.95 (0.82-1.10) | 0.88 (0.72-1.06) | 0.75 (0.56-1.02) | 1.06 (0.80-1.39) | 1.07 (0.85-1.34) |
|  | Q3 | 100-206 | 364 | 0.87 (0.75-1.01) | 0.90 (0.77-1.04) | 0.86 (0.70-1.04) | 0.83 (0.61-1.12) | 0.90 (0.68-1.20) | 0.97 (0.76-1.23) |
|  | Q4 | 207-400 | 394 | 0.83 (0.71-0.96) | 0.87 (0.74-1.01) | 0.82 (0.67-1.00) | 0.91 (0.67-1.23) | 0.74 (0.55-1.00) | 0.94 (0.74-1.20) |
|  | Q5 | ≥401 | 377 | 0.77 (0.65-0.90) | 0.80 (0.68-0.95) | 0.75 (0.60-0.93) | 0.69 (0.50-0.96) | 0.82 (0.60-1.13) | 0.90 (0.69-1.16) |
|  | *P*-trend | |  | 0.001 | 0.009 | 0.02 | 0.14 | 0.08 | 0.21 |
|  | Per 200 g/day - uncalibrated | | |  | 0.94 (0.89-0.98) | 0.93 (0.87-0.99) | 0.93 (0.85-1.02) | 0.93 (0.85-1.02) | 0.95 (0.88-1.02) |
|  |  |  |  |  |  |  |  |  |  |
| **Cheese** | | |  |  |  |  |  |  |  |
|  |  | <5 | 233 | 1.00 | 1.00 | 1.00 | 1.00 | 1.00 | 1.00 |
|  | Q1 | 5-18 | 471 | 1.00 (0.85-1.18) | 0.99 (0.84-1.17) | 0.96 (0.77-1.18) | 0.81 (0.60-1.11) | 1.12 (0.82-1.54) | 1.06 (0.81-1.38) |
|  | Q2 | 19-31 | 464 | 1.00 (0.85-1.19) | 1.02 (0.86-1.21) | 0.97 (0.78-1.21) | 0.83 (0.60-1.15) | 1.08 (0.78-1.50) | 1.10 (0.84-1.45) |
|  | Q3 | 32-54 | 395 | 0.85 (0.71-1.02) | 0.87 (0.73-1.05) | 0.86 (0.68-1.08) | 0.71 (0.50-1.00) | 0.89 (0.63-1.26) | 0.90 (0.68-1.20) |
|  | Q4 | ≥55 | 337 | 0.82 (0.68-1.00) | 0.85 (0.70-1.03) | 0.79 (0.61-1.02) | 0.74 (0.51-1.08) | 0.76 (0.52-1.11) | 0.94 (0.70-1.28) |
|  | *P*-trend | |  | 0.005 | 0.02 | 0.03 | 0.19 | 0.02 | 0.27 |
|  | Per 50 g/day - uncalibrated | | |  | 0.94 (0.86-1.02) | 0.95 (0.85-1.06) | 0.96 (0.80-1.14) | 0.86 (0.72-1.02) | 0.92 (0.80-1.06) |
|  |  |  |  |  |  |  |  |  |  |
| **Yoghurt** | | |  |  |  |  |  |  |  |
|  |  | 0 | 569 | 1.00 | 1.00 | 1.00 | 1.00 | 1.00 | 1.00 |
|  | Q1 | <10.9 | 373 | 0.94 (0.80-1.10) | 0.95 (0.82-1.11) | 1.00 (0.82-1.23) | 1.13 (0.83-1.55) | 0.91 (0.68-1.23) | 0.87 (0.68-1.10) |
|  | Q2 | 11-38 | 299 | 0.81 (0.69-0.94) | 0.84 (0.72-0.99) | 0.97 (0.80-1.19) | 1.00 (0.73-1.37) | 0.88 (0.66-1.18) | 0.68 (0.53-0.87) |
|  | Q3 | 39-101 | 292 | 0.78 (0.67-0.92) | 0.83 (0.71-0.97) | 0.99 (0.81-1.21) | 1.20 (0.90-1.61) | 0.85 (0.64-1.14) | 0.62 (0.48-0.80) |
|  | Q4 | ≥102 | 367 | 0.85 (0.74-0.99) | 0.92 (0.79-1.07) | 1.00 (0.82-1.21) | 1.15 (0.85-1.54) | 0.87 (0.66-1.16) | 0.81 (0.64-1.02) |
|  | *P*-trend | |  | 0.12 | 0.59 | 0.99 | 0.43 | 0.51 | 0.39 |
|  | Per 100 g/day - uncalibrated | | |  | 1.02 (0.96-1.07) | 1.03 (0.96-1.11) | 1.09 (0.99-1.20) | 0.98 (0.88-1.10) | 0.99 (0.91-1.08) |
|  |  | | |  |  |  |  |  |  |
| **Total dairy** | | |  |  |  |  |  |  |  |
|  | Q1 | <115 | 423 | 1.00 | 1.00 | 1.00 | 1.00 | 1.00 | 1.00 |
|  | Q2 | 115-221 | 359 | 0.83 (0.72-0.96) | 0.85 (0.74-0.99) | 0.80 (0.66-0.96) | 0.63 (0.47-0.86) | 0.87 (0.66-1.13) | 0.94 (0.76-1.18) |
|  | Q3 | 222-331 | 370 | 0.78 (0.68-0.91) | 0.83 (0.72-0.96) | 0.79 (0.65-0.96) | 0.73 (0.54-0.97) | 0.82 (0.63-1.08) | 0.89 (0.71-1.12) |
|  | Q4 | 332-525 | 389 | 0.78 (0.67-0.91) | 0.84 (0.72-0.98) | 0.82 (0.67-0.99) | 0.89 (0.67-1.20) | 0.71 (0.53-0.96) | 0.88 (0.69-1.12) |
|  | Q5 | ≥526 | 359 | 0.71 (0.61-0.83) | 0.76 (0.65-0.90) | 0.70 (0.57-0.87) | 0.66 (0.48-0.91) | 0.70 (0.51-0.96) | 0.86 (0.67-1.11) |
|  | *P*-trend | |  | <0.001 | 0.006 | 0.008 | 0.15 | 0.02 | 0.26 |
|  | Per 400 g/day - uncalibrated | | |  | 0.88 (0.81-0.96) | 0.87 (0.78-0.98) | 0.90 (0.76-1.06) | 0.86 (0.72-1.01) | 0.89 (0.78-1.02) |

† Basic model - Cox regression using total energy intake (continuous), and stratified by age (1-year categories), and centre.
 ‡ Multivariable model - Cox regression using total energy intake (continuous), body mass index (continuous), physical activity index (inactive, moderately inactive, moderately active, active, or missing), smoking status and intensity (never; current , 1-15 cigarettes per day; current, 16-25 cigarettes per day; current, 16+ cigarettes per day; former, quit ≤10 years; former, quit 11-20 years; former, quit 20+years; current, pipe/cigar/occasional; current/former, missing; unknown), education status (none, primary school completed, technical/professional school, secondary school, longer education including university, or not specified), alcohol consumption (yes or no; and continuous) and intakes of red and processed meat and fibre (both continuous), and stratified by age (1-year categories), and centre.
 * Total number of colorectal cancer cases across intake categories.

**Table S2.** Multivariable hazard ratios (95% confidence intervals) of colorectal cancer risk in women by dairy product consumption categories.

| **WOMEN** | | | | |  | **Basic model** | **Multivariable model** | **Multivariable models** | | | |
| --- | --- | --- | --- | --- | --- | --- | --- | --- | --- | --- | --- |
|  |  | |  | |  |  |  |  | **Colon cancer** |  |  |
| **Food group (g/day)** | | | | | **CRC cases (n) *** | **Colorectal cancer †** | **Colorectal cancer ‡** | **All colon ‡** | **Proximal ‡** | **Distal ‡** | **Rectal cancer ‡** |
|  |  | |  | |  | **(n = 2,613)** | **(n = 2,613)** | **(n = 1,752)** | **(n = 820)** | **(n = 746)** | **(n = 861)** |
| **Total milk** | | | | |  |  |  |  |  |  |  |
|  | Q1 | | <5.3 | | 469 | 1.00 | 1.00 | 1.00 | 1.00 | 1.00 | 1.00 |
|  | Q2 | | 5.3-82.3 | | 523 | 0.96 (0.84-1.10) | 0.95 (0.84-1.09) | 0.94 (0.80-1.10) | 0.94 (0.74-1.20) | 0.94 (0.73-1.20) | 0.98 (0.78-1.23) |
|  | Q3 | | 82.4-171 | | 491 | 0.93 (0.81-1.06) | 0.92 (0.81-1.06) | 0.96 (0.82-1.13) | 0.96 (0.75-1.22) | 0.92 (0.72-1.18) | 0.85 (0.67-1.08) |
|  | Q4 | | 172-302 | | 568 | 0.88 (0.77-1.00) | 0.88 (0.77-1.00) | 0.90 (0.76-1.06) | 0.84 (0.66-1.07) | 0.97 (0.75-1.24) | 0.84 (0.66-1.06) |
|  | Q5 | | ≥303 | | 262 | 0.82 (0.71-0.94) | 0.81 (0.71-0.93) | 0.82 (0.69-0.97) | 0.88 (0.69-1.13) | 0.78 (0.60-1.02) | 0.79 (0.62-1.01) |
|  | *P*-trend | | | |  | 0.002 | 0.002 | 0.02 | 0.32 | 0.08 | 0.03 |
|  | Per 200 g/day - uncalibrated | | | | |  | 0.94 (0.90-0.99) | 0.94 (0.89-0.99) | 0.96 (0.89-1.04) | 0.94 (0.86-1.02) | 0.95 (0.88-1.03) |
|  |  | |  | |  |  |  |  |  |  |  |
| **Cheese** | | | | |  |  |  |  |  |  |  |
|  |  | | <5 | | 262 | 1.00 | 1.00 | 1.00 | 1.00 | 1.00 | 1.00 |
|  | Q1 | | 5-18 | | 583 | 0.88 (0.76-1.02) | 0.87 (0.75-1.01) | 0.83 (0.69-0.99) | 0.73 (0.57-0.95) | 1.02 (0.76-1.37) | 0.97 (0.74-1.27) |
|  | Q2 | | 19-33 | | 661 | 0.97 (0.83-1.13) | 0.97 (0.83-1.13) | 0.91 (0.75-1.10) | 0.82 (0.63-1.07) | 1.01 (0.75-1.37) | 1.10 (0.83-1.45) |
|  | Q3 | | 34-55 | | 584 | 0.89 (0.76-1.04) | 0.89 (0.75-1.04) | 0.85 (0.70-1.03) | 0.70 (0.53-0.92) | 1.07 (0.79-1.46) | 0.98 (0.74-1.32) |
|  | Q4 | | ≥56 | | 523 | 0.88 (0.74-1.05) | 0.87 (0.73-1.04) | 0.82 (0.67-1.02) | 0.70 (0.52-0.95) | 1.01 (0.73-1.41) | 0.98 (0.72-1.34) |
|  | *P*-trend | | | |  | 0.26 | 0.24 | 0.23 | 0.10 | 0.96 | 0.73 |
|  | Per 50 g/day - uncalibrated | | | | |  | 0.95 (0.88-1.02) | 0.93 (0.85-1.02) | 0.86 (0.75-0.99) | 1.01 (0.88-1.16) | 0.98 (0.86-1.12) |
|  |  | |  | |  |  |  |  |  |  |  |
| **Yoghurt** | | | | |  |  |  |  |  |  |  |
|  |  | | 0 | | 505 | 1.00 | 1.00 | 1.00 | 1.00 | 1.00 | 1.00 |
|  | Q1 | | 0.1-21.3 | | 530 | 1.01 (0.88-1.16) | 1.01 (0.88-1.16) | 0.96 (0.81-1.13) | 0.96 (0.75-1.22) | 0.98 (0.76-1.26) | 1.13 (0.88-1.45) |
|  | Q2 | | 21.4-54.8 | | 518 | 1.00 (0.88-1.14) | 1.01 (0.89-1.15) | 0.97 (0.83-1.14) | 0.94 (0.75-1.18) | 0.99 (0.77-1.26) | 1.11 (0.87-1.40) |
|  | Q3 | | 54.9-112 | | 562 | 0.97 (0.85-1.10) | 0.99 (0.87-1.13) | 0.95 (0.81-1.11) | 0.89 (0.70-1.12) | 1.01 (0.79-1.28) | 1.10 (0.87-1.40) |
|  | Q4 | | ≥113 | | 498 | 0.85 (0.74-0.97) | 0.87 (0.76-1.00) | 0.82 (0.69-0.97) | 0.81 (0.64-1.03) | 0.85 (0.65-1.09) | 1.00 (0.78-1.28) |
|  | *P*-trend | | | |  | 0.002 | 0.015 | 0.013 | 0.06 | 0.17 | 0.50 |
|  | Per 100 g/day - uncalibrated | | | | |  | 0.97 (0.92-1.02) | 0.96 (0.90-1.02) | 0.95 (0.87-1.04) | 0.97 (0.89-1.07) | 0.98 (0.90-1.07) |
|  | | | | | |  |  |  |  |  |  |
| **Total dairy** | | | |  | |  |  |  |  |  |  |
|  | Q1 | <142 | | 529 | | 1.00 | 1.00 | 1.00 | 1.00 | 1.00 | 1.00 |
|  | Q2 | 142-231 | | 505 | | 0.93 (0.82-1.05) | 0.93 (0.82-1.06) | 0.91 (0.78-1.06) | 0.80 (0.64-1.01) | 0.94 (0.75-1.18) | 0.99 (0.80-1.22) |
|  | Q3 | 232-332 | | 515 | | 0.86 (0.76-0.97) | 0.87 (0.76-0.98) | 0.87 (0.75-1.02) | 0.83 (0.67-1.04) | 0.88 (0.70-1.11) | 0.85 (0.68-1.06) |
|  | Q4 | 333-479 | | 492 | | 0.74 (0.65-0.85) | 0.75 (0.66-0.86) | 0.75 (0.64-0.88) | 0.75 (0.60-0.95) | 0.76 (0.60-0.98) | 0.75 (0.59-0.94) |
|  | Q5 | ≥479 | | 359 | | 0.76 (0.66-0.86) | 0.76 (0.67-0.87) | 0.75 (0.64-0.89) | 0.76 (0.60-0.96) | 0.75 (0.58-0.97) | 0.78 (0.62-0.98) |
|  | *P*-trend |  | |  | | <0.001 | <0.001 | <0.001 | 0.05 | 0.013 | 0.011 |
|  | Per 400 g/day - uncalibrated | | |  | |  | 0.88 (0.81-0.95) | 0.86 (0.78-0.95) | 0.90 (0.78-1.04) | 0.85 (0.73-0.99) | 0.92 (0.80-1.05) |

† Basic model - Cox regression using total energy intake (continuous), and stratified by age (1-year categories), and centre.
‡ Multivariable model - Cox regression using total energy intake (continuous), body mass index (continuous), physical activity index (inactive, moderately inactive, moderately active, active, or missing), smoking status and intensity (never; current , 1-15 cigarettes per day; current, 16-25 cigarettes per day; current, 16+ cigarettes per day; former, quit ≤10 years; former, quit 11-20 years; former, quit 20+ years; current, pipe/cigar/occasional; current/former, missing; unknown), education status (none, primary school completed, technical/professional school, secondary school, longer education including university, or not specified), ever use of contraceptive pill (yes, no, or unknown), ever use of menopausal hormone therapy (yes, no, or unknown), menopausal status (premenopausal, postmenopausal, perimenopausal/unknown menopausal status, or surgical postmenopausal), alcohol consumption (yes or no; and continuous) and intakes of red and processed meat and fibre (both continuous), and stratified by age (1-year categories), and centre.
* Total number of colorectal cancer cases across intake categories.

**Table S3.** Multivariable hazard ratios (95% confidence intervals) of colorectal cancer risk in men by dietary calcium intake categories.

| **MEN** | |  | |  | **Basic model** | **Multivariable model** | **Multivariable models** | | | |
| --- | --- | --- | --- | --- | --- | --- | --- | --- | --- | --- |
|  |  |  | |  |  |  |  | **Colon cancer** |  |  |
|  | | | | **CRC cases (n) *** | **Colorectal cancer †** | **Colorectal cancer ‡** | **All colon ‡** | **Proximal ‡** | **Distal ‡** | **Rectal cancer ‡** |
|  |  |  | |  | **(n = 1,900)** | **(n = 1,900)** | **(n = 1,116)** | **(n = 478)** | **(n = 520)** | **(n = 784)** |
| **Calcium (mg/day)** | | | |  |  |  |  |  |  |  |
|  | Q1 | <689 | | 427 | 1.00 | 1.00 | 1.00 | 1.00 | 1.00 | 1.00 |
|  | Q2 | 690-882 | | 408 | 0.91 (0.79-1.04) | 0.94 (0.82-1.08) | 1.00 (0.83-1.20) | 1.04 (0.78-1.39) | 0.97 (0.75-1.25) | 0.86 (0.69-1.08) |
|  | Q3 | 883-1080 | | 376 | 0.83 (0.72-0.96) | 0.89 (0.76-1.03) | 0.91 (0.75-1.10) | 1.01 (0.75-1.37) | 0.83 (0.63-1.11) | 0.85 (0.68-1.08) |
|  | Q4 | 1080-1348 | | 368 | 0.81 (0.69-0.94) | 0.87 (0.74-1.03) | 0.80 (0.65-0.99) | 0.87 (0.63-1.21) | 0.73 (0.53-0.99) | 0.98 (0.77-1.25) |
|  | Q5 | ≥1348 | | 321 | 0.72 (0.61-0.86) | 0.81 (0.67-0.97) | 0.83 (0.65-1.06) | 0.93 (0.65-1.35) | 0.74 (0.52-1.05) | 0.77 (0.58-1.03) |
|  | *P*-trend | | |  | <0.001 | 0.021 | 0.045 | 0.48 | 0.037 | 0.23 |
|  | Per 200 mg/day - uncalibrated | | | |  | 0.95 (0.92-0.98) | 0.96 (0.92-0.99) | 0.95 (0.90-1.01) | 0.95 (0.90-1.01) | 0.94 (0.90-0.99) |
|  |  |  | |  |  |  |  |  |  |  |
| **Dairy calcium (mg/day)** | | | |  |  |  |  |  |  |  |
|  | Q1 | <293 | | 433 | 1.00 | 1.00 | 1.00 | 1.00 | 1.00 | 1.00 |
|  | Q2 | 293-458 | | 376 | 0.82 (0.72-0.95) | 0.85 (0.74-0.98) | 0.84 (0.70-1.01) | 0.72 (0.54-0.97) | 0.93 (0.71-1.21) | 0.87 (0.70-1.08) |
|  | Q3 | 458-637 | | 381 | 0.81 (0.70-0.93) | 0.85 (0.74-0.99) | 0.87 (0.72-1.05) | 0.90 (0.68-1.20) | 0.88 (0.66-1.15) | 0.83 (0.66-1.05) |
|  | Q4 | 638-876 | | 372 | 0.77 (0.67-0.90) | 0.83 (0.71-0.97) | 0.82 (0.67-0.99) | 0.82 (0.61-1.11) | 0.77 (0.57-1.04) | 0.84 (0.67-1.07) |
|  | Q5 | ≥876 | | 334 | 0.72 (0.62-0.85) | 0.78 (0.66-0.92) | 0.77 (0.62-0.96) | 0.72 (0.51-1.01) | 0.76 (0.55-1.06) | 0.80 (0.61-1.04) |
|  | *P*-trend | | |  | <0.001 | 0.01 | 0.034 | 0.15 | 0.07 | 0.13 |
|  | Per 200 mg/day - uncalibrated | | | |  | 0.95 (0.92-0.98) | 0.96 (0.92-0.99) | 0.95 (0.90-1.01) | 0.95 (0.89-1.01) | 0.94 (0.90-0.99) |
|  |  |  |  | |  |  |  |  |  |  |
| **Non-dairy calcium (mg/day)** | | |  | |  |  |  |  |  |  |
|  | Q1 | <308 | 378 | | 1.00 | 1.00 | 1.00 | 1.00 | 1.00 | 1.00 |
|  | Q2 | 309-378 | 444 | | 1.08 (0.93-1.25) | 1.10 (0.94-1.28) | 1.23 (1.00-1.50) | 1.29 (0.95-1.75) | 1.27 (0.95-1.71) | 0.94 (0.75-1.19) |
|  | Q3 | 379-449 | 396 | | 0.97 (0.82-1.14) | 1.01 (0.85-1.19) | 1.09 (0.87-1.37) | 1.03 (0.73-1.45) | 1.23 (0.89-1.71) | 0.90 (0.69-1.16) |
|  | Q4 | 450-544 | 371 | | 0.94 (0.78-1.12) | 0.99 (0.81-1.19) | 1.08 (0.84-1.39) | 0.99 (0.67-1.46) | 1.21 (0.83-1.76) | 0.87 (0.64-1.16) |
|  | Q5 | ≥545 | 307 | | 0.89 (0.72-1.11) | 0.94 (0.75-1.20) | 0.97 (0.71-1.32) | 0.96 (0.59-1.54) | 1.09 (0.69-1.74) | 0.92 (0.64-1.32) |
|  | *P*-trend | |  | | 0.12 | 0.35 | 0.42 | 0.43 | 0.98 | 0.63 |
|  | Per 200 mg/day - uncalibrated | | | |  | 1.00 (0.89-1.12) | 1.05 (0.90-1.22) | 0.96 (0.76-1.20) | 1.15 (0.93-1.43) | 0.94 (0.78-1.13) |

† Basic model - Cox regression using total energy intake (continuous), and stratified by age (1-year categories), and centre.
‡ Multivariable model - Cox regression using total energy intake (continuous), body mass index (continuous), physical activity index (inactive, moderately inactive, moderately active, active, or missing), smoking status and intensity (never; current , 1-15 cigarettes per day; current, 16-25 cigarettes per day; current, 16+ cigarettes per day; former, quit ≤10 years; former, quit 11-20 years; former, quit 20+ years; current, pipe/cigar/occasional; current/former, missing; unknown), education status (none, primary school completed, technical/professional school, secondary school, longer education including university, or not specified), alcohol consumption (yes or no; and continuous) and intakes of red and processed meat, fibre plus mutual adjustment for other dietary calcium source (all continuous), and stratified by age (1-year categories), and centre.
* Total number of colorectal cancer cases across intake categories.

**Table S4.** Multivariable hazard ratios (95% confidence intervals) of colorectal cancer risk in women by dietary calcium intake categories.

| **WOMEN** | | |  | **Basic model** | **Multivariable model** | **Multivariable models** | | | |
| --- | --- | --- | --- | --- | --- | --- | --- | --- | --- |
|  |  |  |  |  |  |  | **Colon cancer** |  |  |
|  | | | **CRC cases (n)** | **Colorectal cancer †** | **Colorectal cancer ‡** | **All colon ‡** | **Proximal ‡** | **Distal ‡** | **Rectal cancer ‡** |
|  |  |  |  | **(n = 2,613)** | **(n = 2,613)** | **(n = 1,752)** | **(n = 820)** | **(n = 746)** | **(n = 861)** |
| **Calcium (mg/day)** | | |  |  |  |  |  |  |  |
|  | Q1 | <650 | 532 | 1.00 | 1.00 | 1.00 | 1.00 | 1.00 | 1.00 |
|  | Q2 | 651-832 | 515 | 0.89 (0.78-1.01) | 0.90 (0.79-1.02) | 0.91 (0.78-1.05) | 0.94 (0.76-1.18) | 0.85 (0.67-1.07) | 0.88 (0.71-1.10) |
|  | Q3 | 833-1010 | 520 | 0.83 (0.73-0.95) | 0.85 (0.74-0.97) | 0.84 (0.71-0.98) | 0.80 (0.63-1.01) | 0.93 (0.73-1.18) | 0.87 (0.69-1.09) |
|  | Q4 | 1011-1251 | 524 | 0.78 (0.68-0.89) | 0.80 (0.70-0.92) | 0.80 (0.67-0.94) | 0.73 (0.56-0.94) | 0.79 (0.61-1.02) | 0.81 (0.63-1.04) |
|  | Q5 | ≥1252 | 522 | 0.72 (0.62-0.84) | 0.75 (0.64-0.87) | 0.71 (0.59-0.87) | 0.76 (0.57-1.00) | 0.69 (0.51-0.93) | 0.81 (0.62-1.07) |
|  | *P*-trend | |  | <0.001 | <0.001 | <0.001 | 0.026 | 0.015 | 0.16 |
|  | Per 200 mg/day - uncalibrated | | |  | 0.95 (0.93-0.98) | 0.94 (0.91-0.98) | 0.95 (0.90-0.99) | 0.96 (0.91-1.01) | 0.97 (0.92-1.02) |
|  |  |  |  |  |  |  |  |  |  |
| **Dairy calcium (mg/day)** | | |  |  |  |  |  |  |  |
|  | Q1 | <315 | 534 | 1.00 | 1.00 | 1.00 | 1.00 | 1.00 | 1.00 |
|  | Q2 | 316-464 | 511 | 0.89 (0.79-1.01) | 0.90 (0.79-1.02) | 0.91 (0.78-1.06) | 0.83 (0.67-1.05) | 1.08 (0.87-1.35) | 0.88 (0.71-1.09) |
|  | Q3 | 465-614 | 509 | 0.84 (0.74-0.95) | 0.85 (0.75-0.96) | 0.85 (0.73-0.99) | 0.89 (0.71-1.11) | 0.81 (0.63-1.03) | 0.84 (0.67-1.05) |
|  | Q4 | 615-824 | 508 | 0.77 (0.68-0.88) | 0.78 (0.69-0.89) | 0.79 (0.67-0.92) | 0.77 (0.61-0.97) | 0.81 (0.63-1.04) | 0.77 (0.61-0.97) |
|  | Q5 | ≥825 | 550 | 0.77 (0.67-0.88) | 0.78 (0.67-0.89) | 0.75 (0.63-0.90) | 0.75 (0.58-0.96) | 0.83 (0.63-1.08) | 0.82 (0.65-1.05) |
|  | *P*-trend | |  | <0.001 | <0.001 | 0.001 | 0.03 | 0.049 | 0.12 |
|  | Per 200 mg/day - uncalibrated | | |  | 0.95 (0.92-0.98) | 0.94 (0.91-0.97) | 0.94 (0.89-0.99) | 0.96 (0.91-1.01) | 0.97 (0.92-1.02) |
|  |  |  |  |  |  |  |  |  |  |
| **Non-dairy calcium (mg/day)** | | |  |  |  |  |  |  |  |
|  | Q1 | <266 | 495 | 1.00 | 1.00 | 1.00 | 1.00 | 1.00 | 1.00 |
|  | Q2 | 267-331 | 572 | 1.07 (0.94-1.23) | 1.11 (0.96-1.27) | 1.08 (0.91-1.27) | 1.08 (0.84-1.38) | 1.07 (0.84-1.37) | 1.16 (0.92-1.48) |
|  | Q3 | 332-394 | 568 | 1.07 (0.92-1.24) | 1.11 (0.96-1.30) | 1.10 (0.91-1.32) | 1.26 (0.96-1.65) | 0.97 (0.73-1.28) | 1.15 (0.88-1.50) |
|  | Q4 | 395-481 | 493 | 0.96 (0.82-1.13) | 1.02 (0.86-1.21) | 1.04 (0.84-1.29) | 1.23 (0.90-1.67) | 0.87 (0.63-1.20) | 0.98 (0.72-1.33) |
|  | Q5 | ≥482 | 484 | 1.01 (0.84-1.22) | 1.10 (0.89-1.35) | 1.12 (0.87-1.45) | 1.32 (0.91-1.92) | 0.93 (0.63-1.38) | 1.06 (0.73-1.53) |
|  | *P*-trend | |  | 0.62 | 0.77 | 0.57 | 0.15 | 0.46 | 0.77 |
|  | Per 200 mg/day - uncalibrated | | |  | 1.03 (0.93-1.14) | 1.07 (0.94-1.21) | 1.12 (0.94-1.34) | 1.01 (0.82-1.24) | 0.95 (0.79-1.15) |

† Basic model - Cox regression using total energy intake (continuous), and stratified by age (1-year categories), and centre.
‡ Multivariable model - Cox regression using total energy intake (continuous), body mass index (continuous), physical activity index (inactive, moderately inactive, moderately active, active, or missing), smoking status and intensity (never; current , 1-15 cigarettes per day; current, 16-25 cigarettes per day; current, 16+ cigarettes per day; former, quit ≤10 years; former, quit 11-20 years; former, quit 20+ years; current, pipe/cigar/occasional; current/former, missing; unknown), education status (none, primary school completed, technical/professional school, secondary school, longer education including university, or not specified), ever use of contraceptive pill (yes, no, or unknown), ever use of menopausal hormone therapy (yes, no, or unknown), menopausal status (premenopausal, postmenopausal, perimenopausal/unknown menopausal status, or surgical postmenopausal), alcohol consumption (yes or no; and continuous) and intakes of red and processed meat, fibre plus mutual adjustment for other dietary calcium source (all continuous), and stratified by age (1-year categories), and centre.
* Total number of colorectal cancer cases across intake categories.
